# Artificial Intelligence in Bacteriophage Science: A Comprehensive Narrative Review of Applications, Challenges, and Translational Opportunities

**DOI:** 10.3390/antibiotics15070635

**Published:** 2026-06-25

**Authors:** Jamil Allen G. Fortaleza, Kevin Smith P. Cabuhat, Herminiño C. Lagunzad, Warren B. Panizales, Jowi Tsidkenu Pili Cruz, Joel G. Matamis, Jose Edwardo R. Mamaat, Amelda C. Libres, Rich Milton R. Dulay, Jose Jurel M. Nuevo

**Affiliations:** 1 National University, Manila 1008, Philippines; hclagunzad@nu-fairview.edu.ph (H.C.L.); wbpanizales@nu-fairview.edu.ph (W.B.P.); 2 Department of Biology, College of Science, De La Salle University, Manila 1004, Philippines; kevin_smith_cabuhat@dlsu.edu.ph (K.S.P.C.); jowi.cruz@dlsu.edu.ph (J.T.P.C.); richmiltondulay@clsu.edu.ph (R.M.R.D.); 3 Basic Education Department, La Consolacion University Philippines, Malolos 3000, Philippines; 4 School of Engineering and Technology, NU Fairview Incorporated, Quezon City 1118, Philippines; 5 School of Medical Laboratory Sciences, St. Dominic College of Asia, Bacoor 4102, Philippines; jgmatamis@sdca.edu.ph; 6 Department of Medical Technology, Far Eastern University, Manila 1015, Philippines; jemamaat@feu.edu.ph; 7 College of Medical Laboratory Science, Liceo de Cagayan University, Cagayan de Oro City 9000, Philippines; alibres@liceo.edu.ph; 8 Center for Tropical Mushroom Research and Development, Central Luzon State University, Science City of Muñoz 3120, Philippines; 9 College of Medical Laboratory Science, Our Lady of Fatima University, Valenzuela City 1440, Philippines; jmnuevo@fatima.edu.ph

**Keywords:** synthetic biology, phage optimization, genome annotation, antimicrobial resistance, machine learning

## Abstract

Antimicrobial resistance and persistent biofilm-associated infections have renewed interest in bacteriophages as alternatives or complements to conventional antibiotics. However, broader therapeutic adoption remains constrained by slow phage discovery, incomplete genome characterization, narrow host range, complex therapeutic matching, and manufacturing variability. Artificial intelligence (AI) offers computational approaches that may help address several of these limitations. This comprehensive narrative review discusses current AI applications across the bacteriophage pipeline, including metagenomic phage discovery, genome annotation, phage–host interaction prediction, personalized phage selection, cocktail optimization, and phage–antibiotic combination design. The review also examines AI-assisted synthetic biology approaches, including receptor-binding protein redesign, CRISPR-enabled engineering, generative genome design, and biosafety screening, as well as emerging applications in bioprocess optimization, yield prediction, purification analytics, quality assurance, and supply-chain management. Current evidence suggests that AI may accelerate phage identification, improve host-range prediction, support therapeutic optimization, and strengthen manufacturing consistency, potentially facilitating the transition of phage therapy from individualized rescue interventions toward more scalable antimicrobial platforms. Nevertheless, major limitations remain, including fragmented, taxonomically biased datasets; limited external validation; restricted interpretability; privacy concerns; biosafety oversight; and evolving regulatory frameworks. Future progress will depend on standardized datasets, multimodal validation, scalable manufacturing systems, experimental and clinical verification, and coordinated regulatory development.

## 1. Introduction

Antimicrobial resistance (AMR) has emerged as one of the most serious threats to global health, progressively reducing the effectiveness of antibiotics that underpin modern medicine. Recent estimates suggest that bacterial AMR contributes to millions of deaths annually, with especially severe burdens in low- and middle-income countries where surveillance systems, diagnostics, and access to advanced therapeutics remain limited [1]. In response, the World Health Organization has identified priority pathogens, including *Klebsiella pneumoniae*, *Acinetobacter* spp., *Escherichia coli*, *Salmonella Typhi*, and *Shigella* spp., all of which exhibit increasing resistance to frontline therapies [2,3]. These trends indicate that AMR is not only a current clinical emergency but also a long-term developmental challenge. Despite the scale of this crisis, antibiotic innovation has not progressed rapidly enough to counter accelerating bacterial resistance. Many newly introduced antibacterial agents remain derivatives of existing drug classes rather than compounds with fundamentally new mechanisms capable of overcoming established resistance pathways [4,5].

This innovation gap has renewed scientific and clinical interest in bacteriophages, or phages, as biologically distinct antibacterial agents. Phages are viruses that specifically infect bacteria, enabling selective targeting of pathogenic organisms while largely sparing beneficial microbiota and mammalian cells [6,7]. Unlike conventional drugs, lytic phages can replicate at the site of infection, thereby increasing their local concentration as long as susceptible bacterial hosts remain present [8,9]. Many phages also encode enzymes such as lysins and depolymerases that can disrupt protective biofilms associated with chronic and device-related infections [10,11]. These properties make phages attractive not only as alternatives to antibiotics but also as complementary tools in difficult-to-treat infections. However, their broader therapeutic adoption has historically been constrained by major translational and operational barriers. Effective treatment often requires rapid matching of the appropriate phage to a patient’s infecting bacterial strain, while host range may be narrow, manufacturing standards remain complex, and regulatory pathways continue to evolve in many jurisdictions [12,13,14]. In addition, the clinical evidence base, although growing, remains less standardized than that of conventional pharmaceuticals. Consequently, phage therapy has often functioned as a customized rescue strategy rather than a scalable mainstream treatment platform [15].

Artificial intelligence (AI) now offers a potential means of addressing many of these longstanding limitations. Advances in sequencing, imaging, proteomics, microbial phenotyping, and digital health systems have generated biological datasets that are too large and complex for traditional analytical methods alone [16,17]. Machine-learning systems can identify hidden patterns within these datasets, enabling faster prediction of resistance genes, treatment outcomes, and microbial behavior [18]. Deep-learning and foundation models further enhance performance by learning transferable representations across genomic, proteomic, and clinical data modalities [19,20]. As a result, AI is increasingly being explored as a strategic enabler of next-generation antimicrobial innovation. Within phage science, AI has potential applications across the full translational pipeline. In discovery, it can mine metagenomic datasets to identify previously unknown phages hidden within viral “dark matter” and prioritize candidates for laboratory validation [21,22,23]. In host prediction, computational models may rapidly infer which phages are most likely to infect a patient’s bacterial isolate, thereby reducing delays associated with empirical screening. In treatment design, AI may optimize phage cocktails, model synergy with antibiotics, and anticipate bacterial resistance evolution before clinical failure occurs [24,25]. In manufacturing, predictive analytics may improve yield, purity control, and supply-chain responsiveness for phage-based products.

Nevertheless, this emerging ecosystem also faces important scientific, practical, and governance challenges. Many AI systems in phage research are trained on fragmented, biased, or relatively small datasets that may limit generalizability across species, regions, and clinical settings [26,27]. Experimental validation often lags behind computational claims, creating a persistent gap between in silico promise and real-world performance. Black-box models may also be difficult to trust in bedside decision-making, where transparency and accountability are essential. Issues of genomic privacy, data ownership, biosafety, and equitable access further complicate implementation [28,29]. At the same time, phage therapy itself continues to face practical barriers, including rapid phage selection, host-specificity constraints, resistance emergence, dosing uncertainty, manufacturing complexity, and regulatory limitations. As summarized in Table 1, AI may help address many of these longstanding obstacles through predictive analytics, optimized phage matching, adaptive cocktail design, personalized dosing models, and scalable quality-control systems.

Accordingly, this narrative review critically examines the integration of artificial intelligence across the bacteriophage translational pipeline, including phage discovery, genome intelligence, host-range prediction, therapeutic optimization, and bioprocess engineering. The review further evaluates the current evidence base, translational limitations, and regulatory challenges associated with AI-enabled phage systems, while assessing their potential to advance phage therapy from individualized rescue interventions toward scalable precision antimicrobial platforms.

## 2. Phage Biology

Bacteriophages replicate primarily through two canonical life strategies, the lytic cycle and lysogeny, each with distinct ecological and translational implications. In the lytic cycle, phages adsorb to specific bacterial surface receptors, inject their genomes, redirect host metabolism toward virion production, and ultimately lyse the bacterial cell to release progeny particles [40,41]. This strategy favors rapid reproductive output and efficient horizontal transmission, allowing lytic phages to suppress bacterial populations through density-dependent predation and to alter microbial community composition [42]. Because immediate bacterial killing is central to therapeutic efficacy, lytic phages are generally preferred in clinical applications. By contrast, lysogeny is a persistence-oriented strategy in which the phage genome integrates into, or is maintained alongside, the bacterial chromosome as a prophage and is replicated during host cell division [42,43]. This enables phage survival during periods of low host density or environmental stress, but it also introduces biological complexities that may limit direct therapeutic use.

The distinction between these strategies is not merely reproductive but functional. Lytic phages primarily influence bacterial abundance through direct killing, whereas temperate phages can reshape host biology over longer timescales. Prophages frequently encode accessory genes that alter bacterial phenotype, including virulence factors, stress-response systems, and metabolic functions, a process known as lysogenic conversion [44,45]. Through these mechanisms, prophages may act as important drivers of bacterial adaptation and evolution rather than passive genomic passengers. However, the same capacity for gene transfer raises translational concerns, particularly when temperate phages carry toxins, fitness determinants, or mobile genetic elements.

The transition between lysis and lysogeny is governed by regulatory systems that integrate intracellular physiology with external ecological signals. Host nutrient availability, growth state, and quorum-sensing molecules can influence phage developmental decisions, allowing replication modes to align with environmental opportunity [46,47]. Regulatory proteins such as the cAMP receptor protein link host metabolic state to phage gene expression, favoring lysogeny during nutrient limitation and lytic replication when bacterial hosts are abundant and metabolically active [47]. These findings indicate that phages function as responsive biological entities capable of sensing host conditions rather than acting as passive infectious particles. Such plasticity may help explain the frequent coexistence of lytic and temperate phages within the same microbial habitats.

The ecological consequences of these life-history strategies extend beyond infection alone. Lytic phages regulate bacterial abundance and contribute to nutrient recycling through host–cell lysis, particularly in aquatic ecosystems where viral turnover influences carbon cycling and biogeochemical fluxes [46,48]. In contrast, lysogenic phages accelerate horizontal gene transfer through mechanisms such as lateral transduction, thereby reshaping microbial evolution and expanding adaptive capacity across bacterial populations [49,50]. In structured environments such as biofilms, lysogeny is often favored because genomic integration supports persistence under spatially stable conditions, whereas fluctuating or nutrient-rich environments more strongly favor lytic expansion [45,51]. Prophages may also modulate host behaviors, including biofilm formation, virulence expression, and stress tolerance, blurring conventional boundaries between parasitism and mutualism [44,52].

## 3. Modern Applications of Phages

In medicine, phages are attracting renewed interest as precision antimicrobials for infections refractory to standard therapy, particularly multidrug-resistant (MDR), chronic, and biofilm-associated infections [53,54]. Selective bacterial targeting may preserve commensal microbiota, an important advantage over broad-spectrum antibiotics that often disrupt microbial homeostasis. This feature is especially relevant in wound infections, prosthetic-device infections, respiratory biofilms, and recurrent urinary tract infections, where antibiotic penetration or efficacy may be limited. The therapeutic potential is further strengthened by phage-derived enzymes, such as endolysins, which directly degrade bacterial cell walls, and by combination strategies using phages with antibiotics or nanomaterials to improve bacterial killing and suppress resistance emergence [54,55]. However, clinical implementation remains constrained by a narrow host range, variable pharmacokinetics, immune neutralization, and the limited number of standardized large-scale trials. Thus, medical use is promising but still transitional rather than fully mainstream.

Beyond human health, phages are increasingly relevant to sustainable agriculture and livestock production. Bacterial plant diseases and intensive farming systems have historically depended on substantial antibiotic use, contributing to environmental contamination and resistance selection. Phage-based biocontrol offers a targeted alternative capable of suppressing plant pathogens and reducing bacterial disease burden in animals without exerting the same broad selective pressure associated with antibiotics [56,57,58,59]. Their host specificity aligns well with precision agriculture models that seek pathogen control while preserving beneficial microbial communities in soil, crops, and animal microbiomes. Nevertheless, field deployment can be less predictable than laboratory efficacy because temperature, UV exposure, moisture, and microbial competition may reduce phage persistence. Consequently, agricultural success depends heavily on formulation and delivery strategy.

Food safety represents one of the most commercially mature applications of phage technology. Foodborne bacterial contamination remains a major cause of morbidity and economic loss, while chemical decontamination approaches may alter product quality or leave undesirable residues. Phages can be applied at multiple stages of the production chain, including raw materials, processing environments, packaging surfaces, and ready-to-eat products [57,60]. Importantly, several phage-based products have already received regulatory approval for food processing use, demonstrating that industrial deployment is feasible when safety, efficacy, and manufacturing standards are clearly defined [61]. Compared with therapeutic applications, this sector benefits from lower biological complexity and clearer endpoints, which may explain its faster regulatory progress.

Environmental applications further expand the role of phages beyond direct disease control. In wastewater systems, aquaculture, and contaminated ecosystems, phages can function as natural regulators of bacterial populations, reducing pathogen burden while helping preserve microbial balance [62,63]. Their biodegradability, self-propagation in the presence of target hosts, and selective activity make them attractive for water treatment and bioremediation. However, environmental systems are highly variable, and outcomes may be influenced by host density, water chemistry, microbial diversity, and ecological feedback loops. As a result, environmental phage use often requires site-specific optimization rather than universal deployment models.

The same biological properties that make phages valuable also impose important limitations. The lytic cycle enables rapid bacterial killing through receptor adsorption, intracellular replication, and host–cell lysis [53,63]. Yet this specificity requires accurate matching between phages and susceptible bacterial strains, frequently necessitating phage cocktails to broaden coverage [53,55,60]. Resistance may also emerge through receptor modification, restriction-modification systems, or CRISPR-Cas immunity [64,65]. Unlike static drugs, however, phages retain the capacity to co-evolve in response to bacterial defenses, creating opportunities for adaptive treatment strategies and engineered phage design [54,66].

**Figure 1:**
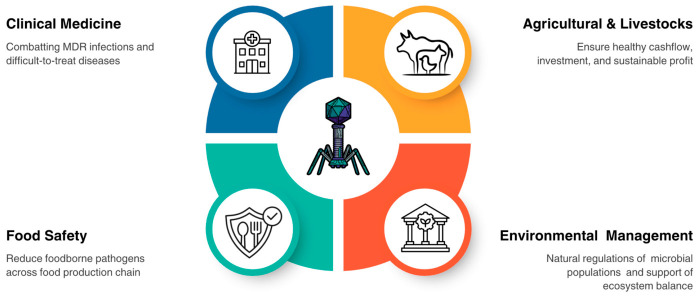
Major modern applications of bacteriophages across diverse sectors.

Despite broad promise, large-scale deployment remains constrained by regulatory, manufacturing, and ecological considerations. Many approval systems are still structured around fixed chemical pharmaceuticals and are not fully adapted to biologically evolving therapeutics [56,59,67]. Manufacturing consistency, formulation stability, and storage under variable conditions remain additional barriers, particularly for commercialization [56,68]. Ecologically, even targeted phage introduction may alter microbial networks or influence horizontal gene transfer if poorly managed [56,62]. As summarized in Figure 1, phages should therefore be viewed not solely as clinical therapeutics, but as multi-sector biological platforms whose future impact will depend on context-specific optimization and scalable governance.

## 4. Translational Barriers to Widespread Phage Adoption

A central limitation is a narrow host range. Most phages recognize highly specific molecular receptors on bacterial surfaces, enabling selective elimination of pathogens while largely sparing beneficial microbiota [69,70,71,72]. In principle, this specificity is therapeutically advantageous because it reduces collateral microbiome disruption. In practice, however, real-world infections are often genetically heterogeneous, and even closely related bacterial strains may differ substantially in susceptibility. Consequently, a single phage frequently fails to provide adequate coverage, particularly in multidrug-resistant (MDR) infections where multiple resistant subpopulations may coexist [70,73]. Current strategies to overcome this problem include phage cocktails, receptor-binding protein engineering, and hybrid constructs designed to broaden host range. Although promising, these approaches depend on a deeper understanding of phage-host interaction networks and bacterial escape evolution, and their reproducibility across pathogens remains inconsistent [69,70,73,74,75]. Thus, host specificity remains both the greatest therapeutic strength and one of the most persistent operational constraints of phage therapy.

Regulatory systems remain similarly misaligned with the biological nature of phage-based interventions. Unlike fixed chemical drugs, phages are replicating biological entities that may evolve over time, making conventional approval pathways difficult to apply. As a result, regulatory frameworks remain fragmented, with different jurisdictions using variable criteria for safety, efficacy, quality control, and clinical access [76,77,78]. This lack of harmonization complicates multinational development, commercialization, and clinician confidence. Although compassionate-use programs and case-based therapeutic successes are increasing, large randomized clinical trials remain comparatively limited, slowing regulatory acceptance, reimbursement models, and routine integration into healthcare systems [61,76,79]. Emerging proposals such as adaptive licensing models and phage-specific guidance frameworks are encouraging, but implementation remains uneven. Consequently, regulatory uncertainty creates both scientific hesitation and commercial risk.

Manufacturing presents an additional and often underestimated bottleneck. Producing phages at scale differs fundamentally from synthesizing small-molecule pharmaceuticals because it requires simultaneous management of both viral and bacterial biological systems. Industrial production involves host cultivation, controlled phage amplification, downstream purification, and rigorous removal of contaminants such as endotoxins, host–cell debris, and residual nucleic acids [37,80,81]. Each step must be tightly standardized to ensure reproducibility, potency, and safety. Stability further complicates deployment, as many phages are sensitive to temperature, pH, and storage conditions, creating challenges in transport, shelf life, and formulation [37,82]. Advances such as encapsulation technologies, improved purification systems, and data-driven quality control may reduce these barriers, yet integration into scalable Good Manufacturing Practice (GMP)-compliant pipelines remains incomplete [37,83].

## 5. AI in Phage Discovery

The historical progression of bacteriophage science, from early empirical discovery to modern AI-assisted innovation, reflects a major transformation in antimicrobial research. In the early twentieth century, bacteriophages were first identified and rapidly explored as therapeutic agents against bacterial infections, although this period preceded computational biology and artificial intelligence [84,85]. While these early efforts demonstrated antimicrobial potential, progress was constrained by limited mechanistic understanding, inconsistent production methods, and the absence of molecular characterization tools. Following the widespread success of antibiotics, phage research declined in many regions, though important expertise persisted in selected centers. Renewed concern over antimicrobial resistance during the 2000s stimulated a global resurgence of interest in phage therapy. At the same time, early bioinformatics tools began supporting genome analysis, taxonomy, and comparative phage studies, allowing the field to move beyond phenotype-based classification toward molecular characterization [85,86,87]. This period was foundational because it established genomic sequencing as a core component of phage discovery, although analytical methods were still heavily dependent on sequence alignment and curated reference databases.

During the 2010s, advances in next-generation sequencing, metagenomics, and computational microbiology generated large-scale datasets that laid the groundwork for machine-learning applications in phage science [22,27,32]. Environmental and clinical metagenomes revealed extensive viral diversity, including large reservoirs of previously uncharacterized sequences often referred to as viral “dark matter”. Conventional homology-based methods were often insufficient for interpreting these data, creating a need for models capable of recognizing patterns beyond direct sequence similarity. Between approximately 2015 and 2020, AI tools increasingly focused on phage-host interaction prediction, host-range specificity, protein structure inference, and functional annotation of previously uncharacterized phage genes [26,30,32,88,89]. Compared with earlier bioinformatics pipelines, these systems offered greater sensitivity for incomplete, divergent, or poorly annotated genomes. However, many early models were trained on relatively limited benchmark datasets, highlighting persistent concerns regarding external validity and biological interpretability.

**Figure 2:**
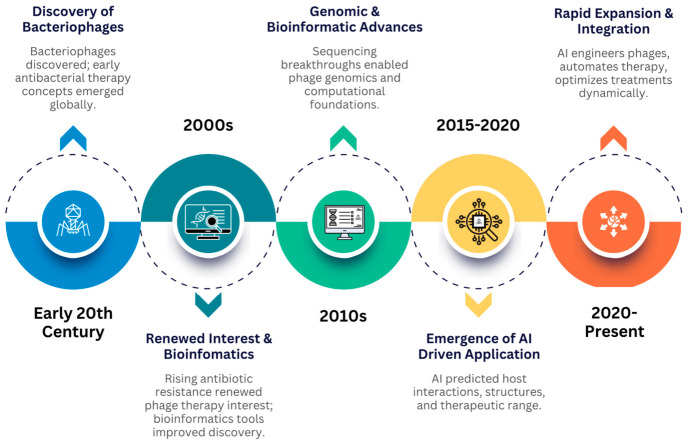
Historical evolution of bacteriophage science toward AI-enabled phage discovery and therapeutic innovation.

Since 2020, AI has expanded from analytical support into translational discovery and design. Current applications include CRISPR-enhanced phages, receptor-binding protein engineering, synthetic genome design, automated therapeutic selection pipelines, and adaptive dosing systems informed by real-time metagenomic feedback. However, many of these approaches require substantial experimental and clinical validation. [22,26,30,32,90,91,92]. These developments suggest that AI is no longer limited to identifying candidate phages but is increasingly involved in optimizing their clinical and industrial utility. Nevertheless, many such approaches remain supported primarily by computational modeling or limited preclinical evidence and require rigorous experimental, translational, and clinical validation. As summarized in Figure 2, the historical progression of phage science illustrates a shift from descriptive microbiology toward precision-engineered discovery systems.

## 6. AI-Driven Phage Discovery and Genome Intelligence

A major application is metagenomic mining. Deep-learning tools such as HVSeeker and MetaPhaPred have shown strong performance in distinguishing phage sequences from bacterial and host DNA. HVSeeker integrates nucleotide- and protein-level information, improving recognition of short or fragmented contigs that are commonly missed by alignment-based approaches [93]. MetaPhaPred combines convolutional neural networks (CNNs) with bidirectional long short-term memory (Bi-LSTM) architectures, enabling simultaneous learning of local sequence motifs and longer-range genomic dependencies associated with phage genomes [94]. These approaches are particularly relevant because many clinically and environmentally important phages remain hidden within mixed metagenomic datasets. However, no single computational strategy is universally optimal for phage detection. As summarized in Table 2, homology-based tools such as VIBRANT v1.2.1 and VirSorter2 v2.2.4 provide strong precision, favorable F1 scores, and robustness to contamination when reference genomes are adequately represented [95]. Their main limitation is reduced sensitivity to highly novel taxa. K-mer-based methods such as Kraken2 offer rapid classification and high precision, but performance may decline in highly diverse communities where sequence composition overlaps across taxa [95]. By contrast, sequence composition-based tools such as DeepVirFinder and Seeker are often more sensitive to phages with limited database representation, although this may occur at the cost of higher false-positive rates or altered diversity estimates [95,96]. In practice, method selection depends on the priority: speed, novelty detection, precision, or ecological profiling.

AI is also helping address the long-standing problem of viral “dark matter,” referring to sequences with no clear homologs or taxonomic assignment. Because many environmental viral genomes cannot be meaningfully annotated through BLAST-based similarity searches alone, self-supervised learning and protein language models provide an important alternative. These models learn structural and evolutionary relationships directly from raw sequence data, enabling functional inference even among highly divergent genomes [97,98]. This marks a conceptual shift in viromics: instead of relying exclusively on known references, computational systems can infer biological relevance from previously unexplored sequence space. Genome annotation is another area where AI has improved performance. Phage genomes are typically compact, gene-dense, and highly mosaic, making accurate annotation difficult for standard microbial gene callers. DeePVP uses deep learning to identify phage virion proteins (PVPs) and classify them into functional categories, with a reported 9.05% increase in F1 score relative to conventional approaches [99]. This is relevant because virion proteins are central to phage assembly, adsorption, host recognition, and infectivity. Complementary frameworks such as METAnnotatorX, together with structure-prediction systems including AlphaFold and ESMFold, enable functional assignment by combining sequence context and predicted three-dimensional structure [100]. Such hybrid strategies are especially useful for proteins lacking detectable sequence homology but retaining conserved structural folds.

**Table 2:** Comparative Analysis of Computational and AI Tools for Phage Detection.

Tool	Approach	Strengths	Limitations	References
VIBRANT	Homology-based	High F1 score (0.93) in the artificial contigs dataset; robust to contamination	May have diversity bias in genome predictions	[95]
VirSorter2	Homology-based	High F1 score (0.93); low false positives; robust to contamination	Similar diversity bias as VIBRANT	[95]
Kraken2	K-mer-based	Highest F1 score (0.86) in mock community; high precision (0.96)	Limited performance in datasets with high diversity	[95]
DeepVirFinder	Sequence composition-based	High sensitivity to phages with low database representation	Higher false positive rates compared with homology-based tools	[96]
Seeker	Sequence composition-based	High sensitivity; capable of detecting diverse phages	Produces genome sets with diversity patterns differing from the original populations	[95,96]
DeePVP	Deep learning	Superior PVP identification (9.05% higher F1 score); reliable predictions	Limited to PVP-specific tasks; requires high-quality input data	[99]

Protein structure prediction has further expanded therapeutic discovery. AI-assisted structural modeling enables faster identification of receptor-binding domains, catalytic residues, and modular architectures in proteins such as tail fibers, lysins, and depolymerases, which are directly relevant to host specificity and antibacterial activity [101]. Likewise, DeepMineLys applies CNN-based learning to identify potent lysins from human microbiome datasets, highlighting how AI can move phage science beyond passive genome cataloging toward active therapeutic candidate prioritization. Despite substantial progress, important limitations remain. HVSeeker may require considerable computational resources [93], while MetaPhaPred depends on sufficiently large and representative training datasets. DeePVP is optimized for task-specific virion protein prediction rather than whole-genome interpretation. AlphaFold may perform less reliably for intrinsically disordered proteins or multimeric assemblies, and DeepMineLys may be sensitive to dataset diversity and validation breadth [102]. More broadly, many current models are trained on curated benchmarks that may not reflect the taxonomic complexity, assembly fragmentation, or ecological diversity of real-world viromes. Accordingly, the next phase of progress will likely depend less on new algorithms alone and more on standardized datasets, multimodal validation, and tighter integration with wet-laboratory workflows.

## 7. AI for Phage-Host Interaction Prediction

**Figure 3:**
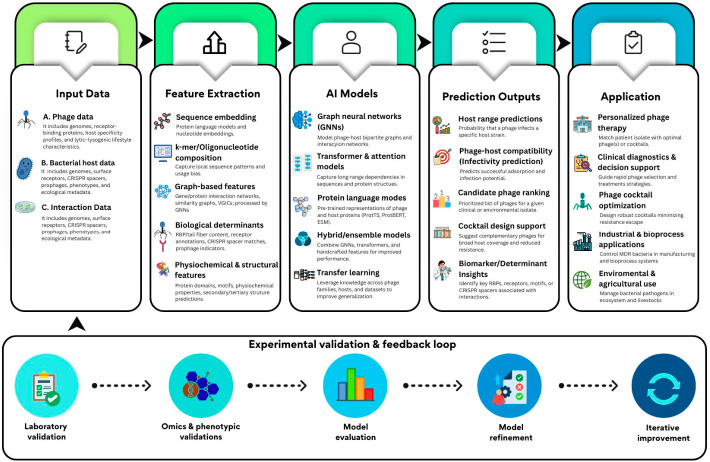
Artificial intelligence framework for phage–host interaction prediction.

Modern AI frameworks now enable high-throughput inference of infectivity, receptor compatibility, and therapeutic host range directly from genomic, proteomic, and network-level data, making them increasingly valuable for precision phage therapy, surveillance microbiology, and microbiome engineering. As illustrated in Figure 3, current prediction pipelines typically begin with diverse data inputs, including phage genomes, bacterial host genomes, prior interaction datasets, and phenotypic information. These inputs are transformed via feature-extraction layers, such as sequence embeddings, protein-domain signals, k-mer composition, biological descriptors, and network relationships, before being processed by predictive models. The resulting outputs may include interaction confidence scores, host-range prediction, receptor-binding compatibility, candidate phage prioritization, cocktail design, and personalized therapeutic selection.

A major methodological advance has been the development of deep-learning systems capable of integrating heterogeneous biological features. Graph neural network (GNN)-based platforms, such as the CM-PHI model, represent phage-bacterium relationships as interaction networks, allowing simultaneous analysis of genomic similarity and topological dependencies that are often missed by alignment-based methods [103]. Similarly, heterogeneous network frameworks such as PHIHNE combine viral and host association data to infer previously unknown links, including experimentally validated predictions [104]. These approaches are significant because host specificity is shaped not only by sequence identity but also by ecological structure, co-evolutionary history, and broader interaction network context. By learning relational biology rather than relying solely on direct homology, network-based AI models can improve predictions for previously unseen phage-host pairs.

Protein language model approaches have introduced an additional layer of predictive power and correspond to the sequence-embedding components. Systems such as GE-PHI use transformer-derived embeddings to encode latent functional information from phage and bacterial proteins, enabling accurate prediction even when closely related reference genomes are absent [105]. Likewise, MoEPH integrates ProtBERT and ProtT5 embeddings with genomic descriptors through a mixture-of-experts framework, achieving reported accuracies of 99.6% on balanced benchmark datasets and a 31% improvement under imbalanced conditions [106]. However, these performance metrics are primarily derived from curated benchmark datasets and should therefore be interpreted cautiously until validated across more diverse real-world settings. These models are particularly relevant because therapeutic datasets are often sparse, noisy, and taxonomically imbalanced. Their performance suggests that protein language representations may become important tools for future host-range prediction.

Also, newer AI models increasingly target strain-level specificity, which is essential for therapeutic deployment. Systems such as PHPGCA and PHISGAE incorporate virus–host similarity learning, adsorption determinants, and host-specific priors to distinguish susceptible from resistant strains within the same bacterial species [107,108,109]. Reported benchmark accuracies approaching 94% highlight the growing feasibility of precision host prediction. This higher resolution is clinically relevant because susceptibility often varies among strains carrying different capsules, efflux systems, CRISPR arrays, restriction systems, or prophage-mediated defenses. Despite promising computational performance, prospective validation across diverse clinical environments remains limited. In practical terms, these models strengthen the host-range prediction stage shown in Figure 3 by narrowing the set of candidate phages prior to empirical screening.

Receptor-binding proteins (RBPs) remain central determinants of host recognition, and AI has become a powerful tool for decoding and engineering these molecules. Deep-learning systems and protein language models can infer structural and biochemical properties of RBPs directly from sequence data, enabling more accurate receptor tropism prediction than motif-based approaches [26]. Transformer-based ProtT5 models reportedly improve weighted F1 scores and recall by approximately 3–4% compared with handcrafted feature strategies [110]. Structure-aware models such as PHIStruct, which combine structural language embeddings with multilayer perceptrons, show 7–9% F1-score improvement when sequence similarity falls below 40%, making them particularly useful for divergent or previously unseen phages [111]. When integrated with structural tools such as AlphaFold, these systems also support rational redesign of tail fibers and adsorption domains to broaden host range or restore activity against resistant strains [112].

## 8. Translational Utility, Benchmarking, and Current Limitations of AI for Phage-Host Interaction Prediction

To address limitations in conventional host prediction, tools such as PHISDetector and DSPHI integrate multiple orthogonal signals, including CRISPR spacer matches, prophage content, oligonucleotide usage bias, defense signatures, and probabilistic reasoning, thereby improving prediction across diverse bacterial populations [113]. PHISDetector has reported species/genus-level accuracies of 51–73% while identifying 85.6% of MDR bacterial hosts, highlighting potential relevance for antimicrobial resistance targeting. Such hybrid systems may be particularly useful in hospital settings where local strain ecology often differs substantially from public database references. However, external validation across geographically diverse healthcare environments remains limited, and predictive performance in polymicrobial infections and heterogeneous clinical samples remains uncertain.

As summarized in Table 3, current AI models differ considerably in architecture, dataset scope, and intended application. CoMPHI and PhageTB demonstrate multi-level taxonomic scalability through hybrid alignment-based and machine-learning approaches, with reported accuracies ranging from 67.9% to 95.1% depending on classification level [114,115]. GSPHI has been optimized for clinically relevant ESKAPE pathogens, achieving 86.65% accuracy and an AUC of 0.9208 [116]. CM-PHI, PHPGCA, and PHIHNE appear particularly useful for identifying previously unseen phage-host interactions. In contrast, MoEPH and PHIStruct demonstrate the growing utility of transformer-based embeddings and structure-aware learning. Nevertheless, no single model is universally optimal, and model selection should depend on the intended application, including clinical triage, ecological surveillance, broad host-range screening, or receptor-level engineering.

Clinically, AI-driven phage-host prediction may support antimicrobial resistance management by prioritizing candidate phages against MDR pathogens and reducing the time required for empirical screening [117]. Additional tools such as PHISDetector and MI-RGC further suggest potential applications extending beyond acute infection treatment into microbiome modulation and ecosystem engineering [118]. AI systems may also support rational phage cocktail design by identifying complementary host ranges and reducing potential resistance escape pathways.

Despite rapid progress, important limitations remain. Many current models still rely on benchmark datasets that may be small, taxonomically biased, or experimentally inconsistent, thereby reducing generalizability in real-world settings. Predictive performance may also decline when applied to novel strains, plasmid-rich genomes, or polymicrobial communities. In addition, limited biological interpretability in some AI systems may affect clinical trust and regulatory acceptance. Future progress will therefore require larger standardized datasets, prospective validation studies, explainable AI architectures, and stronger integration with experimental susceptibility testing.

## 9. AI in Precision Phage Therapy

### 9.1. Predicting Resistance Evolution

Models trained on bacterial genomic signatures and phage activity datasets have demonstrated useful predictive capacity, including successful prioritization of phages for *E. coli* urinary tract infections [119]. Deep learning and protein language models further improve host-range prediction by analyzing receptor-binding proteins, tail fibers, and adsorption-associated domains that determine phage specificity at fine taxonomic resolution [26,120]. Emerging adaptive systems may also combine metagenomic sequencing with reinforcement learning to dynamically modify phage cocktails as resistance evolves [22].

Operationally, AI-assisted workflows integrated with rapid diagnostics may shorten therapeutic timelines. Predictive systems have been reported to reduce actionable treatment times by nearly 29 h compared with conventional culture-based approaches, although most available evidence remains derived from controlled experimental studies [121]. Additional approaches, including Siamese neural networks and algorithm-supported matching systems, have demonstrated promising precision in host-phage prediction and early therapeutic applications [31,122].

Despite these advances, broader clinical implementation remains challenging. Personalized phage therapy requires sequencing infrastructure, susceptibility testing, formulation logistics, immune-response monitoring, and repeated reassessment. Neutralizing antibodies and patient-specific pharmacokinetics may further reduce therapeutic efficacy despite favorable in vitro matching [123,124]. In addition, many current datasets remain limited and potentially biased, while regulatory frameworks for adaptive AI-guided phage systems continue to evolve [22,125]. Consequently, the greatest current value of AI-driven phage selection may lie in improving diagnostic integration and therapeutic prioritization rather than replacing conventional microbiological workflows.

### 9.2. AI for Phage Cocktail Optimization

A primary objective of AI-guided phage cocktail optimization is the selection of complementary phages with overlapping but non-identical host ranges. Network-based models, host-range matrices, and phage-bacteria infection network (PBIN) analyses help identify phages that collectively broaden bacterial coverage while minimizing unnecessary overlap in activity [126,127]. This is particularly relevant in heterogeneous infections where strain-level diversity may reduce the effectiveness of individual phages. AI frameworks may also prioritize receptor complementarity by selecting phages that target distinct bacterial surface structures, thereby reducing the likelihood that a single resistance mutation compromises the entire cocktail.

Beyond expanding coverage, resistance suppression remains a major design objective. Bacterial escape from one phage may impose trade-offs such as slower growth, reduced virulence, or restored susceptibility to other phages within the cocktail. AI-assisted models may exploit these evolutionary constraints by identifying combinations that restrict adaptive escape pathways [128]. Phage-antibiotic synergy (PAS) further extends this concept, where optimized combinations enhance bacterial killing while reducing resistance emergence. Such strategies have demonstrated promising activity against *K. pneumoniae* and other multidrug-resistant pathogens in both in vitro and in vivo studies [129,130,131]. AI may also facilitate synergy discovery by evaluating large numbers of phage-phage and phage-antibiotic interactions that would be difficult to assess experimentally at scale.

Mechanistic pharmacokinetic-pharmacodynamic (PK/PD) models add another layer by simulating phage replication, bacterial growth, immune clearance, and cross-resistance to inform dosing and timing strategies [132]. PBIN analyses may further support the selection of generalist phages for resistant strains while specialist phages fill narrower ecological niches [127]. However, many AI-guided cocktail optimization systems remain supported primarily by computational modeling or limited experimental validation. Excessive cocktail complexity may also increase inter-phage competition, manufacturing difficulty, and regulatory burden. Consequently, although AI-assisted cocktail optimization shows considerable promise, broader clinical implementation will require further translational and prospective validation.

### 9.3. AI-Guided Combination with Antibiotics

A major objective of AI-guided phage-antibiotic optimization is the identification of combinations that maximize bacterial killing while minimizing resistance emergence. Not all phage-antibiotic interactions are beneficial; some combinations exhibit synergistic effects, whereas others remain neutral or antagonistic. Machine-learning systems trained on bacterial growth dynamics, resistance-associated genomic markers, and pharmacokinetic/pharmacodynamic (PK/PD) datasets can help predict pairings most likely to improve therapeutic efficacy under clinically relevant conditions [133,134]. Physics-informed neural networks and hybrid mechanistic frameworks further expand this capability by integrating phage replication kinetics, bacterial population dynamics, and host immune responses to generate more individualized treatment schedules rather than fixed empirical regimens [22].

Therapeutic timing also plays an important role in treatment outcome. Simultaneous phage-antibiotic administration may impose dual selective pressure, accelerating bacterial clearance while limiting adaptive escape. In contrast, early antibiotic exposure may suppress bacterial metabolic activity to levels that reduce phage replication efficiency, thereby weakening synergistic interactions [135]. AI-assisted simulation platforms are particularly valuable in this context because they can rapidly evaluate large numbers of dosing and timing scenarios that would otherwise be difficult to assess experimentally. Dose architecture may similarly influence efficacy, as bolus phage administration can rapidly reduce bacterial density while allowing local phage amplification at the infection site [136,137]. However, many current optimization systems remain supported primarily by controlled experimental datasets and computational simulations, while prospective clinical validation remains limited.

Additional synergy may arise from complementary biological mechanisms. Certain antibiotics disrupt bacterial membranes or weaken biofilm matrices, thereby enhancing phage penetration into otherwise protected bacterial populations. Phages, in turn, may target dormant subpopulations, disrupt biofilm-associated niches, or remove plasmids carrying antimicrobial resistance determinants [138,139,140]. In some cases, phage infection may alter bacterial surface receptors, partially restoring antibiotic susceptibility [135]. These layered interactions help explain why combined therapy may outperform either modality alone, particularly in chronic or device-associated infections where biofilm tolerance is prominent [133,141].

AI systems may also help model adaptive evolutionary trade-offs by identifying therapeutic combinations in which resistance to one agent increases susceptibility to another. Mutations that block phage adsorption may reduce bacterial virulence or increase membrane permeability, whereas certain antibiotic resistance pathways may unintentionally expose new phage targets. The addition of phage cocktails may further restrict the evolutionary routes available for bacterial survival [135,140,142]. Nevertheless, substantial translational barriers remain. Definitions of synergy are not yet standardized, regulatory pathways for biologic-drug combinations remain complex, and host-phage interactions may vary considerably across strains, tissues, immune conditions, and polymicrobial infections [22,35,140]. In addition, external validation across diverse clinical environments remains limited, and many AI-guided optimization systems have not yet been evaluated in large-scale prospective clinical studies. Consequently, although AI-guided phage-antibiotic therapy demonstrates considerable promise, broader implementation will require stronger experimental validation.

## 10. AI + Synthetic Biology + Engineered Phages

### 10.1. CRISPR-Enhanced Phages

AI-assisted CRISPR-enhanced bacteriophages have emerged as a promising strategy that integrates synthetic biology with machine learning to improve precision antimicrobial therapy [133,143,144,145,146,147]. These engineered phages are designed to deliver CRISPR-Cas systems into bacterial cells, enabling sequence-specific targeting of antimicrobial resistance genes, virulence determinants, and plasmid-associated elements. Upon delivery, CRISPR-mediated cleavage may induce lethal genomic damage or eliminate resistance plasmids, potentially restoring antibiotic susceptibility in multidrug-resistant pathogens, such as carbapenem-resistant *E. coli*, while minimizing disruption to commensal microbiota [143,145,146].

Artificial intelligence contributes substantially to the optimization of these systems. Machine-learning approaches can assist in guiding RNA spacer selection, prediction of target accessibility, identification of conserved resistance loci, and reduction in off-target editing events. AI frameworks may also help forecast likely bacterial escape mutations, thereby supporting iterative redesign of CRISPR targets before therapeutic failure occurs. In parallel, AI-guided engineering of receptor-binding proteins, capsid architecture, and genome packaging regions is being explored to improve payload delivery efficiency and expand host range beyond the limitations of naturally occurring phages [144,148]. Additional synthetic strategies, including toxin-antitoxin systems such as CreTA, may further reduce survival of partially edited bacterial populations and strengthen antibacterial efficacy [149].

Despite these advances, major translational challenges remain. Many engineered phages continue to exhibit narrow host specificity, while delivery efficiency may be reduced by serum complement activity, immune neutralization, or poor tissue penetration. Bacterial defense mechanisms, including spacer loss, anti-CRISPR proteins, receptor mutation, and intracellular defense pathways, may also compromise long-term therapeutic activity, necessitating continuous redesign of both phage genomes and CRISPR targets [148]. Additional concerns involve ecological off-target effects, biosafety monitoring, immunogenicity, manufacturing scalability, and the lack of clearly established regulatory pathways for genetically modified phage therapeutics [145,150,151,152,153].

Importantly, many AI-enabled CRISPR-phage systems remain at the proof-of-concept, experimental, or preclinical stage. Current evidence is still dominated by computational studies, laboratory validation, and limited animal-model investigations, while large-scale clinical validation remains scarce. Furthermore, external validation across diverse bacterial species, polymicrobial infections, and heterogeneous clinical environments remains limited. Consequently, although AI-assisted CRISPR-enhanced phages demonstrate considerable therapeutic potential, their real-world clinical applicability and long-term safety remain incompletely established. Future progress will likely depend on tighter integration of AI-guided design, experimental validation, scalable manufacturing, and standardized translational evaluation frameworks. Hybrid delivery systems such as lipid nanoparticles and outer membrane vesicles may further improve delivery efficiency and therapeutic feasibility [145,151]. To improve clarity regarding translational readiness, Table 4 summarizes the current validation stages of AI-enabled CRISPR-enhanced phage systems across computational, experimental, preclinical, and clinical levels.

### 10.2. Generative AI for Genome Design

Recurrent neural networks, long short-term memory (LSTM) models, and transformers have been used to redesign phage genomes, producing variants with expanded host range against *Pseudomonas aeruginosa* and other clinically important pathogens [148]. Larger generative systems, such as megaDNA, further suggest the feasibility of constructing de novo phage genomes containing functional genes and regulatory elements, potentially enabling on-demand therapeutic phage design [154]. A major target of generative AI is the RBP, which governs bacterial recognition and host specificity. Structural prediction systems such as AlphaFold, together with sequence-based neural models, can redesign RBPs to recognize alternative receptors, thereby expanding host range or restoring infectivity against resistant strains [26,155,156]. AI is also being applied to lytic proteins such as lysins, where coevolutionary and generative models have predicted mutations that improve enzymatic activity, thermal stability, and antibacterial potency [157,158]. However, phage genomes are tightly integrated biological systems, and optimizing one trait may compromise assembly efficiency, replication timing, or adsorption kinetics. A broad host range may also trade off against infectivity strength or long-term evolutionary stability. Resistance through receptor mutation, CRISPR defenses, or abortive infection systems remains an additional challenge [26,148]. Consequently, AI-generated phages still require high-throughput functional screening, adsorption assays, burst-size measurements, biofilm testing, and in vivo validation before translation. Ethical concerns, including ecological disruption, horizontal gene transfer, and dual-use misuse of genome design platforms, also require close oversight [26,159].

### 10.3. Safety Optimization

Therapeutic phages are generally preferred to be strictly lytic, since temperate phages may integrate into bacterial chromosomes and facilitate horizontal gene transfer. AI-driven genomic screening now enables rapid identification of integrases, repressors, excisionases, and other lysogeny-associated modules, allowing exclusion or redesign of phages with prophage-like behavior [26,30]. Similar machine-learning and comparative genomics pipelines can detect virulence factors, toxin genes, and cryptic antimicrobial resistance determinants that may be overlooked by conventional annotation methods [22,30]. In this context, AI functions not simply as a screening tool but as a gatekeeper for safer therapeutic phage selection. Ecological containment is another critical dimension of biosafety. Because engineered phages may interact with non-target bacteria or microbial communities, predictive systems such as MVPHI are increasingly used to estimate host range and phage-bacteria interactions before deployment [160]. These models can help minimize unintended effects on beneficial microbiota and reduce environmental dissemination risks. AI-guided sequence optimization may also identify recombination hotspots, unstable repeats, packaging conflicts, or mutation-prone regions that could compromise genomic stability or enable unanticipated evolution after release [30,158].

Immunogenicity is increasingly relevant, particularly for repeated-dosing or chronic-infection protocols. AI-assisted epitope mapping and deimmunization strategies may reduce antibody recognition while preserving infectivity and therapeutic persistence [158]. However, computational predictions alone are insufficient. In vivo validation remains essential to assess immune responses, biodistribution, persistence, toxicity, and therapeutic efficacy under physiological conditions. High-throughput sequencing, proteomic profiling, infectivity studies, and animal models, therefore, remain necessary complements to in silico screening [161]. Real-time metagenomic surveillance may further support adaptive monitoring during therapy by detecting resistance emergence or ecological perturbation [22]. Regulatory implications are substantial, as engineered phages occupy a hybrid space between biologics, gene-delivery systems, and adaptive therapeutics. AI can streamline biosafety compliance through automated genome audits, structured risk documentation, and predictive release testing focused on genomic integrity and contaminant risk [37]. Nevertheless, fragmented international frameworks remain a major barrier to standardized approval.

### 10.4. Smart Programmable Phages

Smart programmable bacteriophages represent a new generation of living therapeutics created through the convergence of synthetic biology and AI. Rather than relying solely on innate host specificity and lytic activity, these engineered phages can sense bacterial quorum signals, metabolic states, inflammatory cues, or biofilm markers, triggering conditional killing only when pathogenic behavior is detected [162,163]. This logic-based targeting may reduce disruption of beneficial microbiota while intensifying pressure on virulent or resistant subpopulations. CRISPR-Cas payloads further extend this concept by enabling phages to remove resistance determinants such as bla-CTX-M or mecA, disrupt virulence genes, or genetically disable pathogens rather than relying only on lysis [164,165]. AI plays a central role by predicting host compatibility, refining circuit thresholds, selecting guide RNAs, and optimizing payload placement within compact phage genomes.

Beyond bacterial killing, programmable phages may function as delivery systems for antimicrobial proteins, biofilm-degrading enzymes, immune modulators, or peptide-display constructs relevant to oncology and targeted drug delivery [166]. However, robustness remains a key challenge because engineered circuits must remain functional during phage replication, mutation, and host switching. AI-assisted redesign, adaptive phage rotation, and multiplexed guide RNA payloads may help preserve efficacy while limiting escape through receptor mutation or anti-CRISPR defenses [142,167]. Containment is equally important. Replication-limited phages, kill-switch systems, and biocontained capsids may reduce ecological persistence and horizontal gene transfer risks, while AI can predict unintended host-range expansion before deployment [165]. Potential clinical applications include MDR infections, microbiome editing, implant-associated biofilms, and adjunctive immunotherapy [168]. Yet regulatory uncertainty, immune clearance, scalable manufacturing, and biosafety validation remain major barriers [169].

## 11. AI in Phage Manufacturing and Quality Control

### 11.1. Bioprocess Optimization

**Figure 4:**
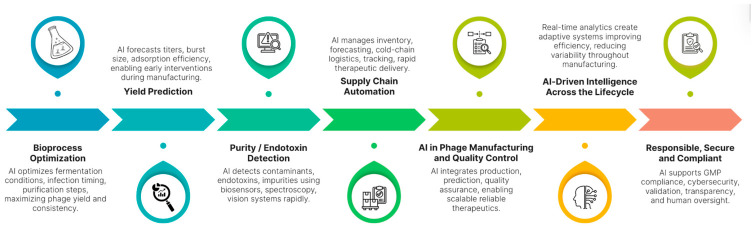
Artificial intelligence applications across the bacteriophage.

In upstream fermentation, variables such as temperature, multiplicity of infection (MOI), agitation, oxygen transfer, and host metabolic state strongly influence phage yield. AI-assisted factorial optimization has demonstrated productivity increases of up to 550-fold, suggesting that infection timing and physiological state may be more important than biomass alone [170]. Real-time sensor systems linked to predictive algorithms can dynamically regulate pH, dissolved oxygen, nutrient feed rates, and agitation to reduce batch failures and improve industrial reproducibility [171,172]. Machine-learning models can also identify the most permissive growth phase for infection, maximizing adsorption efficiency and burst size while minimizing nutrient waste. Downstream processing remains equally important because therapeutic phages require concentration, purification, and stabilization without compromising infective potency or formulation consistency. AI-guided Bayesian optimization and simulation frameworks have improved chromatography, membrane filtration, and separation workflows, with reported yield gains of 18–22% while preserving functional performance [37,173]. These systems are particularly valuable because conventional downstream optimization is often resource-intensive and highly process-specific. AI can translate successful bench-top or pilot conditions into industrial bioreactors by modeling shear stress, aeration gradients, mixing behavior, and oxygen-transfer dynamics, thereby reducing costly scale-up failure [174]. Predictive maintenance, automated control systems, and process anomaly detection may further improve throughput while lowering labor and operational variability. These upstream and downstream functions position AI not as a single manufacturing tool, but as an integrated process controller across multiple production stages (Figure 4).

### 11.2. Yield Prediction

AI-based yield prediction converts this reactive model into a predictive control system. Machine-learning systems can estimate burst size, adsorption efficiency, culture productivity, and final batch titer during production. These models learn from historical fermentation data and identify combinations of conditions associated with optimal phage amplification [37]. Adsorption efficiency, influenced by receptor availability, bacterial density, and physicochemical conditions, can also be predicted from infection kinetics data, allowing early recognition of poorly performing host-phage interactions [80]. Real-time bioreactor signals, such as optical density, dissolved oxygen, pH, and metabolic activity, can act as proxies for infection progression. Time-series models, particularly Long Short-Term Memory (LSTM) networks, are well-suited to these dynamic systems because they capture temporal dependencies and abrupt state changes [132]. For example, sudden oxygen shifts or turbidity decline may indicate premature lysis or delayed infection, both of which strongly affect yield. Classifiers can categorize batches as high-performing, at-risk, or likely to fail using early-stage process data. This enables proactive actions such as adjusting MOI, modifying feed strategies, or terminating underperforming runs to reduce waste [22]. Digital twin systems extend this capability by simulating process changes before implementation in the physical plant [83].

### 11.3. Purity/Endotoxin Detection

AI-assisted purity and endotoxin detection are becoming increasingly important in bacteriophage. Traditional assays, such as Limulus amebocyte lysate testing and culture-based contamination screening, remain valuable, but they are labor-intensive and poorly suited for continuous manufacturing environments. AI, when combined with biosensors, spectroscopy, computer vision, and anomaly detection, shifts purity assurance from retrospective testing toward real-time process intelligence. Biosensor systems such as phage-based biosensors, surface plasmon resonance (SPR), quartz crystal microbalance (QCM), and electrochemical platforms provide rapid detection of contaminants or lysis-associated impurities [175]. Electrochemical impedance systems integrated with multivariate analytics have demonstrated sensitivity below regulatory endotoxin thresholds [176,177]. Spectroscopic approaches, including Raman scattering, fluorescence sensing, and quantum-dot optical systems, further allow label-free and multiplexed monitoring of contaminants and product attributes [178]. Machine-learning tools such as EndoNet can classify endotoxin sources and estimate concentrations across broad dynamic ranges, offering reagent-free alternatives to conventional assays [179]. Computer vision systems can also detect foaming, turbidity shifts, filter breakthrough, or purification anomalies in real time, reducing the risk of full-batch loss [37,180]. However, performance depends heavily on calibration quality, representative training data, and robustness across different phage matrices [179,181].

### 11.4. Supply Chain Automation

A major application is demanding forecasting. Because phage therapy is often linked to multidrug-resistant infections, demand may fluctuate according to hospital outbreaks, antimicrobial resistance trends, and local treatment alternatives. Predictive analytics using microbiology data, prescription trends, and historical utilization can estimate future needs, helping prevent overproduction waste or underproduction delays [182,183]. Inventory management is equally important. Unlike conventional drugs, phage inventories may consist of strain-specific libraries with differing host range, potency, stability, and regulatory status. Machine-learning systems can rank stocks according to predicted clinical utility, expiration risk, and regional pathogen prevalence, enabling more efficient phage-bank management. This is especially relevant for personalized therapy, where treatment success depends on rapid access to matched phages rather than large, undifferentiated inventories.

Cold-chain logistics are another critical area. AI systems integrating IoT sensors, route data, and stability models can detect temperature excursions, estimate remaining product viability, and recommend release, retesting, or discard decisions [182,183]. AI can also optimize rapid hospital delivery, dispatch routes, and nearest-source allocation for urgent infections such as sepsis or persistent bacteremia. Traceability and compliance remain essential. Automated systems can link each batch to genomic screening, sterility, endotoxin testing, storage conditions, and release decisions. Blockchain-assisted traceability may further strengthen auditability and regulatory confidence [77,78]. However, fragmented approval pathways still complicate large-scale deployment [78]. Cybersecurity is equally critical because supply chains depend on sensitive clinical and manufacturing data. Table 5 summarizes the major AI techniques currently applied in phage manufacturing, quality control, logistics, and supply chain management, together with their primary applications and operational roles.

## 12. Challenges and Limitations

### 12.1. Poor and Fragmented Datasets

Although AI-assisted bacteriophage research is frequently described as a data-rich field, one of its primary limitations remains the fragmentation and inconsistency of available datasets. Phage-related information is distributed across genomic repositories, metagenomic surveys, ecological studies, and infection assays that often differ substantially in sequencing quality, annotation depth, metadata structure, and preprocessing workflows. Consequently, phage detection and classification tools based on sequence composition, homology, or machine learning frequently generate inconsistent and only partially overlapping outputs [97,184]. These inconsistencies reflect not only technical variability but also the absence of harmonized standards for phage annotation, taxonomy, and metadata reporting.

Another major challenge is the limited integration of phenotypic and ecological information with genomic datasets. Host-range measurements, adsorption kinetics, resistance profiles, receptor usage, and environmental metadata are frequently sparse, incomplete, or entirely absent, thereby limiting the ability of computational systems to establish reliable genotype-phenotype associations [104]. Independent datasets are also commonly affected by narrow ecological sampling, region-specific collection bias, limited host overlap, inconsistent taxonomy, and variable preprocessing strategies, which collectively reduce interoperability across studies [96,185].

These structural limitations directly influence predictive reliability and cross-study reproducibility. Models trained on fragmented or poorly harmonized datasets may perform well under benchmark conditions yet fail when applied to diverse phage populations, novel bacterial hosts, or broader ecological environments [107,184]. Bias may additionally emerge through overrepresentation of well-studied bacterial species and the scarcity of validated negative interaction datasets, thereby distorting assumptions regarding infectivity and host specificity [96,107]. Variability in preprocessing, annotation criteria, and input formatting may further generate inconsistent outputs despite the use of identical computational frameworks.

Current solutions increasingly emphasize ecosystem-level integration rather than isolated algorithmic refinement. Standardized annotation systems, curated repositories, graph-based learning, multimodal architectures, transfer learning, contrastive learning, and multi-omic integration approaches may help reconcile multi-source datasets into more biologically coherent representations [104,108,186]. Ultimately, future progress will likely depend less on raw sequencing volume and more on the development of interoperable, biologically contextualized, and standardized phage data infrastructures.

### 12.2. Small Sample Sizes

Limited sample size remains one of the most significant barriers in AI-driven bacteriophage research because experimentally validated phage-host interaction datasets are often sparse, strain-specific, and unevenly distributed across biological contexts. In applications such as host-range prediction, receptor recognition, and phage cocktail optimization, the relatively small number of validated interactions reduces statistical robustness and limits the ability of computational models to distinguish biologically relevant patterns from stochastic associations [119,187].

The problem is further amplified by the high dimensionality of phage-related data. Genomic and proteomic prediction systems may evaluate thousands of sequence-derived features, k-mer distributions, structural motifs, or protein domains while relying on comparatively few labeled training examples. Under these conditions, models become highly susceptible to overfitting, dataset-specific noise, and unstable predictive performance across biologically diverse datasets [118,119,188]. In phage display systems, limited sample diversity may additionally amplify experimental artifacts, particularly when amplification bias distorts enrichment patterns and creates misleading signals of functional selection [189,190].

Several computational strategies may partially mitigate these limitations. Transfer learning enables models trained on broader biological datasets to be adapted for phage-specific applications, thereby reducing dependence on extensive labeled datasets [191,192]. Data augmentation approaches, including controlled k-mer transformations, weak labeling, structured perturbation, and synthetic sequence generation using variational autoencoders or related generative models, may further improve training diversity and predictive robustness [187,193,194,195]. However, artificially expanded datasets remain valuable only when they maintain realistic biological patterns and sequence constraints rather than introducing computational artifacts or synthetic bias.

### 12.3. Black-Box Models

Black-box AI systems have substantially accelerated bacteriophage research by improving prediction of host range, phage-host compatibility, therapeutic candidate selection, and infection-associated genomic patterns. However, high predictive accuracy does not necessarily translate into biological understanding. Many deep learning and ensemble-based systems generate outputs with limited explanation regarding why a particular phage is predicted to infect a specific host or display therapeutic potential [107,115,116]. This lack of interpretability becomes especially problematic when computational predictions are used to guide laboratory screening, therapeutic phage prioritization, cocktail design, or clinical decision-making.

In phage biology, mechanistic transparency is particularly important because infectivity is often determined by highly specific molecular interactions involving receptor-binding proteins, tail fibers, depolymerases, adsorption modules, and bacterial surface receptors [26,121,196]. Predictive systems that fail to identify these determinants may therefore provide limited scientific insight despite strong computational performance. Furthermore, some black-box frameworks may rely on shortcuts such as taxonomic similarity, sequence redundancy, or annotation artifacts rather than biologically causal determinants of infectivity, thereby limiting confidence in their translational reliability [31,115,116].

Explainable AI (XAI) has emerged as an important strategy for improving interpretability, biological plausibility, and model auditing. Approaches such as feature attribution, saliency mapping, attention analysis, and domain-importance scoring can identify the genomic or structural features most strongly influencing model predictions [31,197,198]. When these computationally identified features correspond to experimentally relevant mechanisms, they help bridge predictive modeling with testable biological hypotheses [120].

Beyond interpretability, XAI may also support hypothesis generation and experimental prioritization. Predictions linking infectivity to specific motifs, structural domains, or receptor-binding determinants can subsequently be validated through mutagenesis, adsorption assays, structural modeling, or host-range experiments [26,30]. In the long term, interpretable AI systems may facilitate adaptive precision phage therapy frameworks integrating metagenomic surveillance, susceptibility testing, and dynamic therapeutic updating [22]. Ultimately, the scientific value of AI in bacteriophage research will depend not only on predictive performance, but also on its ability to reveal the molecular mechanisms underlying infection, resistance, and phage–host co-evolution.

### 12.4. Biological Validation Gap

A major source of this disconnect lies in the layered complexity of validation systems. Most computational models are trained on simplified datasets, whereas experimental environments progressively reintroduce biological constraints. In vitro assays such as plaque formation, time-kill curves, and biofilm reduction studies provide rapid and scalable first-pass validation, yet they frequently overestimate translational success. Phages that perform strongly on agar plates or in liquid culture may lose efficacy in vivo because of tissue barriers, altered bacterial metabolism, spatial heterogeneity, or immune-mediated neutralization [199,200,201,202]. Detection of phage particles after administration may confirm persistence, but not necessarily productive infection or therapeutic benefit [200].

Animal models and advanced physiological systems provide greater realism, but they also introduce variability in biodistribution, dosing kinetics, inflammation, and host-specific pharmacology that remain difficult to standardize [203,204,205]. What appears as model failure may therefore reflect a mismatch between computational abstraction and biological depth rather than a purely algorithmic flaw.

A second challenge is asymmetry of scale. AI systems can evaluate thousands of genomes, rank host interactions, and generate extensive candidate lists within hours. Experimental validation remains constrained by time, labor, biosafety requirements, and standardized culturing capacity. Tools such as MetaPhage v0.3.3, vHULK v2.0.0, and Phirbo have improved large-scale host prediction and viral classification, yet they infer potential rather than demonstrate infectivity, replication competence, or therapeutic utility [206,207,208]. Consequently, false positives accumulate when predicted compatibility does not yield productive infection, while rare but biologically valuable interactions may never be tested.

Equally important is the temporal nature of phage biology, which static models only partially capture. Host range is dynamic, resistance is nonlinear, and infection outcomes can change rapidly under selective pressure. Predicted susceptibility may disappear through receptor masking, phase variation, CRISPR-mediated defense, or abortive infection systems, while resistant bacteria may regain vulnerability through compensatory trade-offs [26,30,202]. Likewise, cocktails predicted to be synergistic may compete intracellularly or select unforeseen resistance pathways when tested experimentally [209].

A more effective framework would treat computation and experimentation as a recursive system rather than a linear pipeline. AI should generate ranked hypotheses that move through tiered validation platforms: automated in vitro screening, biofilm and organoid models, organ-on-chip systems, and focused in vivo studies [204,206,210]. Each stage should return structured outcome data, such as adsorption success, resistance emergence, immune interference, or pharmacokinetic loss, for model retraining. Real-time imaging, qPCR, single-cell tracking, and immune profiling can further enrich these feedback loops [200,202].

### 12.5. Ethical and Regulatory Barriers

AI-assisted phage applications sit at the intersection of two adaptive domains: biologically evolving therapeutics and algorithmic decision systems. Each independently challenges conventional regulation; together, they expose substantial gaps in existing ethical and legal frameworks. Phage therapy already differs from standard pharmaceuticals because phages may replicate, require personalization, or be reformulated in response to resistance. When AI is layered onto this model for host prediction, cocktail design, resistance forecasting, or manufacturing control, regulatory complexity increases further [30,76,211].

Existing agencies such as the FDA, EMA, and comparable national authorities typically assess therapeutics through frameworks built around reproducibility, batch consistency, and stable indication claims. AI-assisted phage systems complicate all three. A phage cocktail may be updated in response to emerging resistance, while the model selecting that cocktail may also be retrained on new data. This raises a foundational question: is the regulated product the phage preparation, the algorithm, the combined system, or the adaptive workflow itself? Current regulatory structures offer only partial answers [30,76,211].

The challenge extends to clinical evidence generation. Randomized controlled trial designs may be difficult to reconcile with personalized or dynamically updated phage interventions. Ethical tensions arise between evidentiary rigor and compassionate access, particularly for patients with multidrug-resistant infections lacking alternatives [76,212]. Adaptive trials, platform studies, and registry-linked evidence models may therefore become more suitable than conventional fixed-product trials.

Safety evaluation is also dual-layered. Biological risks include lysogeny-associated genes, toxin genes, transduction potential, contamination, and manufacturing purity. Although phage therapy has shown encouraging safety profiles, standardized evaluation remains uneven, especially when candidates are selected or engineered through AI-guided systems [213,214]. Computational risks are equally relevant: false-positive host predictions, poorly calibrated synergy estimates, or opaque optimization systems may lead to ineffective or poorly justified treatment choices [26,30]. In this context, safety must include model validation, uncertainty estimation, dataset quality, and robust human oversight.

Algorithm accountability is therefore central. If a treatment recommendation emerges from a partially opaque model, responsibility for adverse outcomes may be unclear among clinicians, developers, hospitals, manufacturers, and regulators. Explainable AI, structured audits, and post-deployment monitoring can improve accountability by clarifying which features drove recommendations and whether those features were biologically plausible [215,216,217,218,219].

Dual-use concerns add another ethical layer. The same AI tools that optimize host specificity or engineering efficiency for therapeutic purposes could, in principle, be misused to design disruptive biological agents or harmful targeting systems [26,30]. Governance must therefore balance controlled access and proportionate oversight without unnecessarily suppressing beneficial innovation.

A credible response requires multilevel coordination. Principles such as transparency, accountability, fairness, ethics, and safety must be translated into reporting standards, audit requirements, interoperable regulation, and independent review systems [220,221]. The deeper insight is that AI-assisted phage therapy challenges regulation because both components are adaptive systems. Future governance may need to regulate not only products, but the quality and safety of adaptation itself.

### 12.6. Data Ownership and Clinical Privacy

Legal frameworks such as GDPR, HIPAA, and related national privacy regulations strongly influence how patient-derived microbial data can be collected, stored, transferred, and reused. These systems emphasize informed consent, data minimization, anonymization, access control, and accountability, yet cross-border collaboration remains difficult because privacy requirements vary substantially across jurisdictions [222,223,224]. This fragmentation is particularly relevant for phage research, where clinically meaningful datasets may need to be pooled internationally to capture sufficient pathogen diversity, resistance phenotypes, and treatment outcomes.

Consent models present an additional complexity. Traditional one-time consent may be poorly suited to AI-driven workflows in which datasets are repeatedly reused for retraining, external validation, secondary analyses, or future applications that are not fully predictable at the time of collection. More adaptive frameworks, including dynamic consent, tiered consent, and collaborative ownership models such as CHDO, may better preserve patient agency while supporting responsible scientific reuse [225]. In this sense, consent should be viewed not as a one-time administrative event, but as an evolving relationship between patients, institutions, and data ecosystems.

These governance constraints directly affect model quality. When institutions cannot easily share data, AI systems are trained on smaller and less diverse cohorts, increasing the risk of sampling bias, overfitting, and poor generalizability. Anonymization alone is not always sufficient, particularly when genomic, geographic, temporal, and clinical metadata can be recombined to infer identity. Privacy protection, therefore, requires more sophisticated technical safeguards than simple de-identification.

Several privacy-preserving approaches are increasingly relevant. Federated learning allows institutions to train shared models while retaining raw data locally, reducing the need for centralized transfer, although challenges remain in communication cost, model leakage, and cybersecurity resilience [226]. Differential privacy, secure multiparty computation, encryption-based analytics, and blockchain-enabled audit trails may further reduce exposure risk while preserving accountability [227,228]. Decentralized infrastructures such as the Personal Health Train provide an especially useful model by allowing algorithms to travel to local datasets rather than requiring sensitive data to move across institutions [224].

However, privacy solutions must preserve scientific utility. Excessive data restriction or aggressive anonymization may remove clinically meaningful context, including treatment timing, resistance trajectories, ecological source information, or co-morbidity signals that are highly relevant for phage prediction models. The challenge is therefore not maximal privacy or maximal openness, but proportional governance that protects individuals while retaining biological signals.

Cross-institutional progress will depend on governance systems that are technically secure, legally coherent, and socially trustworthy. Shared data-use agreements should define ownership, permitted uses, attribution, benefit-sharing, withdrawal rights, and breach-response responsibilities. International standards aligned with interoperable frameworks may improve collaboration, while ethics boards and access committees should include clinicians, microbiologists, data scientists, legal experts, and patient representatives [227,229].

## 13. Conclusions

The convergence of artificial intelligence and bacteriophage science offers a promising route toward precision antimicrobial therapy in the face of rising resistance. Although phages provide selective bacterial targeting and adaptive potential, their wider adoption has been limited by narrow host range, slow matching workflows, manufacturing complexity, and regulatory uncertainty. AI may help overcome these barriers through faster phage discovery, improved host prediction, optimized cocktail design, resistance forecasting, and smarter clinical decision support. However, current progress remains early-stage. Many AI models rely on limited datasets, retrospective benchmarking, and insufficient real-world validation. Additional challenges include interpretability, biosafety oversight, standardized production, and integration into clinical practice. In the near term, AI is most likely to function as a decision-support tool that accelerates phage selection, laboratory testing, and manufacturing optimization rather than replacing human expertise. If supported by rigorous validation, scalable production, and clear regulatory frameworks, AI-enabled phage therapy could become a practical and important precision antimicrobial strategy in the post-antibiotic era.

## Figures and Tables

**Table 1:** Major Challenges in Phage Therapy and the Potential Role of AI-Enabled Solutions.

Challenges in Phage Therapy	How AI-Enabled Phage Therapy Addresses These Challenges	References
Rapid Phage Selection	AI algorithms can rapidly analyze patient-specific metagenomic and microbiological data to identify the most suitable therapeutic phages from validated phage libraries, improving treatment speed, safety, and efficacy.	[30]
Personalized Dosing Optimization	Physics-informed neural networks (PINNs) and other predictive models simulate phage-bacteria-host dynamics to generate individualized dosing regimens, maximizing therapeutic outcomes while minimizing failure risk.	[22]
Host-Specificity Limitations	AI-based host prediction tools use deep learning and genomic pattern recognition to accurately predict phage-host interactions, enabling precise targeting of bacterial strains.	[31,32]
Emergence of Phage Resistance	AI-driven analytics support the rational design of phage cocktails, sequential therapies, and engineered phages to overcome bacterial resistance and sustain antimicrobial efficacy.	[26,33]
Time-Intensive Diagnostic and Treatment Processes	AI-integrated workflows shorten the diagnosis-to-treatment timeline by combining rapid pathogen identification, real-time metagenomic feedback, and adaptive treatment optimization, potentially reducing delays from days to hours.	[34]
Limited Experimental and Clinical Data	AI helps compensate for limited datasets by using computational modeling for protein structure prediction, genome annotation, transfer learning, and functional inference, accelerating phage discovery and development.	[35,36]
Manufacturing and Quality Control Challenges	Machine-learning models can optimize phage production parameters, predict batch variability, and improve quality assurance processes for scalable therapeutic manufacturing.	[37]
Regulatory and Clinical Translation Barriers	AI-supported evidence synthesis, clinical decision systems, and explainable models may facilitate regulatory evaluation, standardization, and clinician confidence in phage therapy adoption.	[38,39]

**Table 3:** Advanced AI Models for Phage-Host Interaction Prediction.

Model	Features	Methodology	Dataset Scope	Reported Performance	Clinical Relevance	Reference
MoEPH	Integrates transformer-derived protein embeddings (ProtBERT, ProtT5) with statistical genomic descriptors	Mixture-of-experts framework with gated fusion mechanism	Three public benchmark datasets (e.g., 101 hosts, 129 phages)	Accuracy: 99.6% on balanced datasets; 31% improvement under imbalanced conditions	Highly robust under class imbalance; suitable for real-world sparse therapeutic datasets	[106]
PHIStruct	Structure-aware receptor-binding protein (RBP) embeddings using structural language models	Multilayer perceptron (MLP) with SaProt embeddings	ESKAPE genera	7–9% F1-score improvement when sequence similarity < 40%	Excellent for detecting hosts of highly divergent or novel phages	[111]
ProtT5-based Model	Contextual embeddings of RBPs using protein language modeling	Transformer-based sequence encoder	Not specified	3–4% gains in weighted F1 and recall vs. handcrafted features	Strong for functional annotation of RBPs and host-recognition prediction	[110]
CoMPHI	Combines nucleotide/protein encodings with alignment similarity	Hybrid alignment-based + machine-learning framework	Species to phylum taxonomic levels	AUC-ROC: 94.0–96.7%; Accuracy: 92.3–95.1%	High taxonomic scalability across multiple classification levels	[115]
CM-PHI	Multi-hop attention graph neural network + gated convolutional sequence encoder	Integrates topology-level and sequence-level features via self-attention	Heterogeneous microbial interaction network	Superior robustness and accuracy over baseline methods	Strong candidate for predicting unseen phage-host links	[103]
PHPGCA	Virus-virus and virus–host similarity learning with graph augmentation	Graph contrastive learning + LightGCN embeddings	Virus-prokaryote graph datasets	Strong multi-species host prediction performance	Effective for broad host-range screening and ecological prediction	[107]
PHIHNE	Viral-host heterogeneous network mining	Similarity network fusion + graph embedding	Four benchmark datasets	Novel predictions experimentally validated	Integrates network biology with experimentally supported inference	[104]
PHISDetector	Multi-signal PHIS features (CRISPR, prophage, oligonucleotide profiles, defense signals)	Machine-learning ensemble framework	758 annotated phage-host pairs + metagenomic datasets	Accuracy: 51–73% (species/genus); identified 85.6% of MDR bacterial hosts	Valuable for antimicrobial resistance targeting and metagenomic host assignment	[113]
GSPHI	DNA/protein embeddings combined with interaction graph features	SDNE graph embedding + deep neural network	ESKAPE pathogen dataset	Accuracy: 86.65%; AUC: 0.9208	Optimized for clinically important ESKAPE pathogens	[116]
PhageTB	Hybrid alignment-free and alignment-based host prediction	Ensemble framework integrating multiple classifiers	Validation set (1201 interactions)	Accuracy: 67.9–93.5% across taxonomic levels	Flexible multi-level host taxonomy prediction	[114]

**Table 4:** Validation Stages of AI-Enabled CRISPR-Enhanced Phage Systems.

AI-Enabled Phage Strategy	Application	Evidence	Remarks	References
AI-guided CRISPR spacer optimization	Guide RNA design, off-target minimization, and escape mutation prediction	Extensive	No established clinical implementation	[133,143,144]
CRISPR-enhanced bacteriophages	Sequence-specific bacterial killing and resistance plasmid curing	Strong computational support	No established clinical implementation	[145,146,147]
AI-guided receptor-binding protein engineering	Host-range expansion and payload delivery optimization	Strong computational support	No established clinical implementation	[144,148]
CreTA-integrated CRISPR systems	Suppression of partially edited bacterial populations	Computationally supported	No established clinical implementation	[149]
Hybrid delivery systems (lipid nanoparticles and outer membrane vesicles)	Enhanced CRISPR-phage delivery and stability	Primarily conceptual/computational	Limited preclinical investigation	[145,151]

**Table 5:** Summary of AI Techniques in Phage Manufacturing and Quality Control.

Manufacturing Stage	AI Technique	Function	Remarks	References
Bioprocess Optimization	Machine Learning (Regression, Optimization Algorithms)	Models’ optimal fermentation parameters, such as pH, temperature, and multiplicity of infection (MOI)	Improved consistency and reduced batch variability	[37]
	Reinforcement Learning	Adaptive control of bioreactor conditions	Real-time process optimization	[83]
Yield Prediction	Neural Networks (ANN, LSTM)	Predicts phage titer during production	Early intervention and reduced waste	[22,133]
Quality Control	Machine Learning + Spectral Analysis	Detects contaminants such as endotoxins and host DNA	Increased detection accuracy	[30]
	AI-enabled Biosensors	Real-time endotoxin monitoring	Faster quality assurance	[162]
	Computer Vision	Monitors purification processes	Automated anomaly detection	[162]
Predictive QA	Predictive Modeling	Forecasts contamination risks	Preventive quality control	[30]
Supply Chain Automation	Predictive Analytics	Forecasts demand and production needs	Optimized inventory management	[34]
	Optimization Algorithms	Logistics and distribution planning	Reduced delivery delays	[38]
	Blockchain + AI Integration	Ensures traceability and transparency	Enhanced regulatory compliance	[38]

## Data Availability

No new data were created or analyzed in this study. Data sharing is not applicable to this article.

## References

[1] Gauba A., Rahman K.M. (2023). Evaluation of Antibiotic Resistance Mechanisms in Gram-Negative Bacteria. Antibiotics.

[2] Mukhopadhyay S., Peng Y., Tun H.M. (2025). The 2024 WHO bacterial priority pathogens list: A critical evolution from a global One Health perspective. Sci. One Health.

[3] Sati H., Carrara E., Savoldi A., Hansen P., Garlasco J., Campagnaro E., Boccia S., Castillo-Polo J.A., Magrini E., Garcia-Vello P. (2025). The WHO Bacterial Priority Pathogens List 2024: A prioritisation study to guide research, development, and public health strategies against antimicrobial resistance. Lancet Infect. Dis..

[4] Theuretzbacher U. (2025). Evaluating the innovative potential of the global antibacterial pipeline. Clin. Microbiol. Infect. Off. Publ. Eur. Soc. Clin. Microbiol. Infect. Dis..

[5] Butler M.S., Gigante V., Sati H., Paulin S., Al-Sulaiman L., Rex J.H., Fernandes P., Arias C.A., Paul M., Thwaites G.E. (2022). Analysis of the Clinical Pipeline of Treatments for Drug-Resistant Bacterial Infections: Despite Progress, More Action Is Needed. Antimicrob. Agents Chemother..

[6] Alsayed A.R., Permana A.D. (2024). Bacteriophages Therapy: Exploring Their Promising Role in Microbiome Modulation and Combatting Antibiotic Resistance. OBM Genet..

[7] Kapoor A., Mudaliar S.B., Bhat V.G., Chakraborty I., Prasad A.S.B., Mazumder N. (2024). Phage therapy: A novel approach against multidrug-resistant pathogens. 3 Biotech.

[8] Rohde C., Wittmann J., Kutter E. (2018). Bacteriophages: A Therapy Concept against Multi-Drug-Resistant Bacteria. Surg. Infect..

[9] Harshitha N., Rajasekhar A., Saurabh S., Sonalkar R., Tejashwini M., Mitra S.D. (2022). Bacteriophages: Potential Biocontrol Agents and Treatment Options for Bacterial Pathogens. Clin. Microbiol. Newsl..

[10] Wang M., Zhang J., Wei J., Jiang L., Jiang L., Sun Y., Zeng Z., Wang Z. (2024). Phage-inspired strategies to combat antibacterial resistance. Crit. Rev. Microbiol..

[11] Youssef R.A., Sakr M.M., Shebl R.I., Aboshanab K.M. (2025). Recent insights on challenges encountered with phage therapy against gastrointestinal-associated infections. Gut Pathog..

[12] Fortaleza J.A.G., Ong C.J.N., Jesus R.D. (2024). Efficacy and clinical potential of phage therapy in treating methicillin-resistant Staphylococcus aureus (MRSA) infections: A review. Eur. J. Microbiol. Immunol..

[13] Fortaleza J.A.G., Felisco C.K.C., Shete V., Wangchuk J., Rigby J.J.C.D., Mitra A., Nuevo J.J.M. (2025). Phage therapy for Pseudomonas aeruginosa lung infections: A review. J. Pure Appl. Microbiol..

[14] Fortaleza J.A.G., Cabuhat K.S.P., Kim S., Mortel F.A., Bacalzo G.D., Nuevo J.J.M. (2026). Bacteriophages targeting Acinetobacter baumannii in the era of antibiotic failure: A review. Front. Microbiol..

[15] Cui L., Watanabe S., Miyanaga K., Kiga K., Sasahara T., Aiba Y., Tan X.E., Veeranarayanan S., Thitiananpakorn K., Nguyen H.M. (2024). A Comprehensive Review on Phage Therapy and Phage-Based Drug Development. Antibiotics.

[16] Ali S., Basu S. (2025). Big Data Analytics and Antimicrobial Resistance. Artificial Intelligence in Managing Antimicrobial Resistance.

[17] Mi K., Marini S., Lin Z. (2026). Application of machine learning and artificial intelligence methods for predicting antimicrobial resistance. Machine Learning and Artificial Intelligence in Toxicology and Environmental Health.

[18] Visonà G., Duroux D., Miranda L., Sükei E., Li Y., Borgwardt K., Oliver C. (2023). Multimodal learning in clinical proteomics: Enhancing antimicrobial resistance prediction models with chemical information. Bioinformatics.

[19] Ren Y., Chakraborty T., Doijad S., Falgenhauer L., Falgenhauer J., Goesmann A., Schwengers O., Heider D. (2022). Deep Transfer Learning Enables Robust Prediction of Antimicrobial Resistance for Novel Antibiotics. Antibiotics.

[20] de la Lastra J.M.P., Wardell S.J.T., Pal T., de la Fuente-Nunez C., Pletzer D. (2024). From Data to Decisions: Leveraging Artificial Intelligence and Machine Learning in Combating Antimicrobial Resistance—A Comprehensive Review. J. Med. Syst..

[21] Kolluru V., Hole S.R., Sagar A., Chintakunta A.N., R J., Salotagi S. (2025). AI-Prediction of Neisseria gonorrhoeae Resistance at the Point of Care from Genomic and Epidemiologic Data. Healthcare.

[22] Rahimian M., Mohammadi E., Aghazadeh-Soltan-Ahmadi M., Samari A., Zarghami N. (2026). An update on experimental to large-scale production of bacteriophages against superbugs: A review. Crit. Rev. Biotechnol..

[23] Panagiotopoulos A.P., Sagona A.P., Tsakri D., Ferous S., Anastassopoulou C., Tsakris A. (2025). Virological and Pharmaceutical Properties of Clinically Relevant Phages. Antibiotics.

[24] Mehta N., Nguyen A.T., Rodriguez E.K., Young J. (2025). Smart Phages: Leveraging Artificial Intelligence to Tackle Prosthetic Joint Infections. Antibiotics.

[25] Kaneko T., Nakatsuka K., Tsuneda S. (2026). Phage cocktails: State-of-the-art technologies and strategies for effective design. FEMS Microbiol. Rev..

[26] Xu S., Yang S., Jiao X., Cai J., Wu J., Qiao J. (2026). From structure to design: Experimental and AI-driven approaches in receptor-binding protein engineering for reprogramming phage host range. Arch. Microbiol..

[27] Turner P.E., Azeredo J., Buurman E.T., Green S., Haaber J.K., Haggstrom D., Kameda de Figueiredo Carvalho K., Kirchhelle C., Gonzalez Moreno M., Pirnay J.-P. (2024). Addressing the research and development gaps in modern phage therapy. PHAGE Ther. Appl. Res..

[28] Nouis S.C., Uren V., Jariwala S. (2025). Evaluating accountability, transparency, and bias in AI-assisted healthcare decision- making: A qualitative study of healthcare professionals’ perspectives in the UK. BMC Med. Ethics.

[29] Krejcar O., Abdullah J., Namazi H. (2026). Implementing XAI in life sciences: Key challenges and pathways to solutions. Artif. Intell. Life Sci..

[30] Wu P., Li W., Zhang W., Li S., Deng B., Xu S., Li Z. (2025). Advanced strategies in phage research: Innovations, applications, and challenges. Microorganisms.

[31] Wang X., Fu M., Lin S., Wang Y., Pei H. (2026). A deep Siamese network framework for precision phage selection in pulmonary infections. Front. Med..

[32] Doud M.B., Robertson J.M., Strathdee S.A. (2025). Optimizing phage therapy with artificial intelligence: A perspective. Front. Cell. Infect. Microbiol..

[33] Fujiki J., Yokoyama D., Yamamoto H., Kimura N., Shimizu M., Kobayashi H., Nakamura K., Iwano H. (2025). Biocontrol of Phage Resistance in Pseudomonas Infections: Insights into Directed Breaking of Spontaneous Evolutionary Selection in Phage Therapy. Viruses.

[34] Ahmad M.N., Ahmad S.S., Qureshi A.M. (2026). Revolutionizing Antimicrobial Resistance. Combatting Antimicrobial Resistance Through Medical Microbiology.

[35] Patel R.R., Arun P.P., Singh S.K., Singh M. (2025). Overcoming antimicrobial resistance: Phage therapy as a promising solution to combat ESKAPE pathogens. Int. J. Antimicrob. Agents.

[36] Hein Z.M., Guruparan D., Okunsai B., Che Mohd Nassir C.M.N., Ramli M.D.C., Kumar S. (2025). AI and Machine Learning in Biology: From Genes to Proteins. Biology.

[37] Mohammadi E., Rahimian M., Panahi B. (2025). Bridging the gap: Phage manufacturing processes from laboratory to agri-food industry. Virus Res..

[38] Abbas Q., Jeong W., Lee S.W. (2025). Explainable AI in clinical decision support systems: A meta-analysis of methods, applications, and usability challenges. Healthcare.

[39] Fukaya-Shiba A., Ogata A., Kuribayashi R., Sakurai A., Suzuki K., Takadama S., Nishimura J., Uchiyama J., Ohge H., Takeuchi T. (2025). Regulatory considerations for developing phage therapy medicinal products for the treatment of antimicrobial resistant bacterial infections. Front. Pharmacol..

[40] Gilbert R.A., Kelly W.J., Altermann E., Leahy S.C., Minchin C., Ouwerkerk D., Klieve A.V. (2017). Toward understanding phage-host interactions in the rumen; complete genome sequences of lytic phages infecting rumen bacteria. Front. Microbiol..

[41] Jin Y., Li W., Zhang H., Ba X., Li Z., Zhou J. (2023). The post-antibiotic era: A new dawn for bacteriophages. Biology.

[42] Rollie C., Chevallereau A., Watson B.N.J., Chyou T.-Y., Fradet O., McLeod I., Fineran P.C., Brown C.M., Gandon S., Westra E.R. (2020). Targeting of temperate phages drives loss of type I CRISPR–Cas systems. Nature.

[43] Li P., Yong S., Zhou X., Shen J. (2022). Characterization of a new temperate Escherichia coli phage vB_EcoP_ZX5 and its regulatory protein. Pathogens.

[44] Feiner R., Argov T., Rabinovich L., Sigal N., Borovok I., Herskovits A.A. (2015). A new perspective on lysogeny: Prophages as active regulatory switches of bacteria. Nat. Rev. Microbiol..

[45] Silveira C.B., Rohwer F.L. (2016). Piggyback-the-winner in host-associated microbial communities. npj Biofilms Microbiomes.

[46] Williamson S.J., Paul J.H. (2006). Environmental factors that influence the transition from lysogenic to lytic existence in the φHSIC/Listonella pelagia marine phage-host system. Microb. Ecol..

[47] Laganenka L., Sander T., Lagonenko A., Chen Y., Link H., Sourjik V. (2019). Quorum sensing and metabolic state of the host control lysogeny-lysis switch of bacteriophage T1. mBio.

[48] Ashy R.A., Agustí S. (2020). Low host abundance and high temperature determine switching from lytic to lysogenic cycles in planktonic microbial communities in a tropical sea (Red Sea). Viruses.

[49] Liu Z., Tang K., Zhou Y., Liu T., Guo Y., Wu D., Wang X. (2024). Active prophages in coral-associated Halomonas capable of lateral transduction. ISME J..

[50] Berg M., Goudeau D., Olmsted C., McMahon K.D., Yitbarek S., Thweatt J.L., Bryant D.A., Eloe-Fadrosh E.A., Malmstrom R.R., Roux S. (2021). Host population diversity as a driver of viral infection cycle in wild populations of green sulfur bacteria with long standing virus-host interactions. ISME J..

[51] Morella N.M., Gomez A.L., Wang G., Leung M.S., Koskella B. (2018). The impact of bacteriophages on phyllosphere bacterial abundance and composition. Mol. Ecol..

[52] Basso J.T.R., Ankrah N.Y.D., Tuttle M.J., Grossman A.S., Sandaa R.-A., Buchan A. (2020). Genetically similar temperate phages form coalitions with their shared host that lead to niche-specific fitness effects. ISME J..

[53] Alaaeddine R., Al-Mohammed N.H., Pillai J., Fayyad-Kazan M. (2025). Phage therapy for drug-resistant infections: Mechanisms, evidence, and emerging clinical strategies. Mol. Biol. Rep..

[54] Hwang J., Kim J. (2025). Phage therapy as a modern alternative to antibiotics. Edelweiss Appl. Sci. Technol..

[55] Kosznik-Kwaśnicka K., Necel A., Piechowicz L. (2026). Searching for the perfect match: Can non-antibiotic antimicrobials improve bacteriophage performance?. Front. Cell. Infect. Microbiol..

[56] Baud A., Rougis I., Bertolla F. (2026). A century-old solution for 21st century challenges: Current applications with a focus on biocontrol, environmental impacts, and regulatory perspectives. Antibiotics.

[57] Bhardwaj N., Bhardwaj S.K., Deep A., Dahiya S., Kapoor S. (2015). Lytic bacteriophages as biocontrol agents of foodborne pathogens. Asian J. Anim. Vet. Adv..

[58] Jassim S.A.A., Limoges R.G. (2014). Natural solution to antibiotic resistance: Bacteriophages ‘The Living Drugs’. World J. Microbiol. Biotechnol..

[59] Pathak-Vaidya P., Sharma S., Telang M. (2021). Bacteriophage as an antibacterial agent: A patent perspective. Future Microbiol..

[60] Zhao J., Xiao F., Gui J., Guo D., Yang X., Lin Y., Wang C., Ye H., Thida Maung A., Noor Mohammadi T. (2025). The prospect and challenge of bacteriophages for biocontrol in food industry. Food Rev. Int..

[61] Fokas R., Kalatzis P.G., Vantarakis A. (2026). Bacteriophages as food biocontrol agents: A One Health framework for manufacturing quality, regulatory governance, and ethical stewardship—A narrative review. Viruses.

[62] Rogovski P., Cadamuro R.D., da Silva R., de Souza E.B., Bonatto C., Viancelli A., Michelon W., Elmahdy E.M., Treichel H., Rodríguez-Lázaro D. (2021). Uses of bacteriophages as bacterial control tools and environmental safety indicators. Front. Microbiol..

[63] Bisen M., Kharga K., Mehta S., Jabi N., Kumar L. (2024). Bacteriophages in nature: Recent advances in research tools and diverse environmental and biotechnological applications. Environ. Sci. Pollut. Res..

[64] Wang J., Yu Z., Li J. (2025). LHPre: Phage host prediction with VAE-based class imbalance correction and lyase sequence embedding. IEEE Trans. Comput. Biol. Bioinform..

[65] Rezaei A.R. (2024). Bacteriophages for the treatment of resistant bacterial infectious diseases. J. Bacteriol. Virol..

[66] Chavan R., Purandare K. (2025). Bacteriophage therapy inspired new age technologies to control antimicrobial resistance. J. Umm Al-Qura Univ. Appl. Sci..

[67] Elfadadny A., Ragab R.F., Abou Shehata M.A., Elfadadny M.R., Farag A., Abd El-Aziz A.H., Khalifa H.O. (2024). Exploring bacteriophage applications in medicine and beyond. Acta Microbiol. Hell..

[68] Wang X., Zhang S., Ahn J. (2025). Bridging phage production models and practical applications to control antibiotic-resistant bacteria. Microbiol. Res..

[69] Yosef I., Goren M.G., Globus R., Molshanski-Mor S., Qimron U. (2017). Extending the host range of bacteriophage particles for DNA transduction. Mol. Cell.

[70] Chung K.M., Liau X.L., Tang S.S. (2023). Bacteriophages and their host range in multidrug-resistant bacterial disease treatment. Pharmaceuticals.

[71] de Jonge P.A., Nobrega F.L., Brouns S.J.J., Dutilh B.E. (2019). Molecular and evolutionary determinants of bacteriophage host range. Trends Microbiol..

[72] Ross A., Ward S., Hyman P. (2016). More is better: Selecting for broad host range bacteriophages. Front. Microbiol..

[73] Yao G., Le T., Korn A.M., Peterson H.N., Liu M., Gonzalez C.F., Gill J.J. (2023). Phage Milagro: A platform for engineering a broad host range virulent phage for Burkholderia. J. Virol..

[74] Myers J., Davis J., Lollo M., Hudec G., Hyman P. (2023). More’s the same—Multiple hosts do not select for broader host range phages. Viruses.

[75] Vaiyapuri M., Raveendran K., George I., Gundubilli D., Sivam V., Krishnan S.G., George J.C., Mothadaka M.P., Nagarajarao R.C., Badireddy M.R. (2021). Comparison of single and multi-host enrichment approach for harnessing lytic phages against antimicrobial-resistant E. coli: Repurposing the enrichment step. Biologia.

[76] Fürst-Wilmes M., Respondek V., Lilienthal N., Buss K., Düchting A. (2025). Regulation of phage therapy medicinal products: Developments, challenges and opportunities [Regulierung von Phagenarzneimitteln: Entwicklungen, Herausforderungen und Chancen]. Bundesgesundheitsblatt-Gesundheitsforschung-Gesundheitsschutz.

[77] Moon K., Coxon C., Årdal C., Botgros R., Djebara S., Durno L., Fiore C.R., Perrin J.-B., Dixon D.M., Cavaleri M. (2025). Considerations and perspectives on phage therapy from the transatlantic taskforce on antimicrobial resistance. Nat. Commun..

[78] Fuerst-Wilmes M., Respondek V., Schramm M., Lilienthal N., Buss K., Duechting A. (2025). Regulation of phage therapy medicinal products: Developments, challenges, and opportunities. Front. Cell. Infect. Microbiol..

[79] Pirnay J.-P., Merabishvili M., De Vos D., Verbeken G. (2024). Bacteriophage production in compliance with regulatory requirements. Methods Mol. Biol..

[80] Caffin C., Milhamont L., Duriez E., Hembert A., Huzet P., Lerouge C., Deblieck M., Watier D. (2024). Optimization of bacteriophage propagation in high-yield continuous culture (cellstat) meeting the constraints of industrial manufacturing processes. J. Biosci. Bioeng..

[81] de Souza C.M., Tanir T., Orellana M., Escalante A., Koeris M.S. (2021). Manufacturing bacteriophages (Part 2 of 2): Formulation, analytics and quality control considerations. Pharmaceuticals.

[82] Branston S.D., Stanley E.C., Ward J.M., Keshavarz-Moore E. (2013). Determination of the survival of bacteriophage M13 from chemical and physical challenges to assist in its sustainable bioprocessing. Biotechnol. Bioprocess Eng..

[83] Tanir T., Orellana M., Escalante A., de Souza C.M., Koeris M.S. (2021). Manufacturing bacteriophages (Part 1 of 2): Cell line development, upstream, and downstream considerations. Pharmaceuticals.

[84] Pertics B.Z., Király L., Bozsó Z., Krüzselyi D., Nagy J.K., Künstler A., Samu F., Schwarczinger I. (2026). Phage therapy in plant disease management: 110 years of history, current challenges, and future trends. Plants.

[85] Harper D.R., McConville M., Anderson F.J., Enright M.C. (2014). Antimicrobial phages. Molecular Medical Microbiology.

[86] Rizvi S.M.D., Lila A.S.A., Moin A., Syed S., Khafagy E.-S., Askoura M., Rajab A.A.H., Hegazy W.A.H. (2025). Bacteriophage resurrection: Innovative impacts in medicine, biotechnology, and environmental solutions. Sci. Afr..

[87] Gulig P., Swindle S., Eisenman D. (2025). Emerging use of bacteriophages, natural predators of bacteria, to combat antibiotic resistance in the clinic and beyond: A review of the field and biosafety assessment. Appl. Biosaf..

[88] Nie W., Qiu T., Wei Y., Ding H., Guo Z., Qiu J. (2024). Advances in phage–host interaction prediction: In silico method enhances the development of phage therapies. Brief. Bioinform..

[89] Yang Y., Fan C., Zhao Q. (2020). Recent advances on the machine learning methods in identifying phage virion proteins. Curr. Bioinform..

[90] Yuan X., Fan L., Jin H., Wu Q., Ding Y. (2025). Phage engineering using synthetic biology and artificial intelligence to enhance phage applications in food industry. Curr. Opin. Food Sci..

[91] Getz L.J., Patel P.H., Maxwell K.L. (2025). A solution to the postantibiotic era: Phages as precision medicine. Curr. Opin. Microbiol..

[92] Al-Khafaji Z., Altalbawy F.M.A., Jamil N.Y., Sahib A.S., Abdulali Z.S., Hashem A., Hamodi Z.A., Hamo S.H., Alwan M., Jawad M. (2026). Phage therapy in oncology: Mechanisms, innovations, and translational advances. J. Drug Deliv. Sci. Technol..

[93] Al-Najim A., Hauns S., Tran V.D., Backofen R., Alkhnbashi O.S. (2025). HVSeeker: A deep-learning-based method for identification of host and viral DNA sequences. GigaScience.

[94] Ma L., Deng W., Bai Y., Du Z., Xiao M., Wang L., Li J., Nandi A.K. (2023). Identifying Phage Sequences From Metagenomic Data Using Deep Neural Network With Word Embedding and Attention Mechanism. IEEE/ACM Trans. Comput. Biol. Bioinform..

[95] Fung S., Wheeler N., Millard A., van Schaik W. (2023). Gauge your phage: Benchmarking of bacteriophage identification tools in metagenomic sequencing data. Microbiome.

[96] Schackart K.E., Graham J.B., Ponsero A.J., Hurwitz B.L. (2023). Evaluation of computational phage detection tools for metagenomic datasets. Front. Microbiol..

[97] Flamholz Z.N., Li C., Kelly L. (2024). Improving viral annotation with artificial intelligence. mBio.

[98] Mutz P., Camargo A.P., Sahakyan H., Neri U., Butkovic A., Wolf Y.I., Krupovic M., Dolja V.V., Koonin E.V. (2025). The protein structurome of Orthornavirae and its dark matter. mBio.

[99] Fang Z., Feng T., Zhou H., Chen M. (2022). DeePVP: Identification and classification of phage virion proteins using deep learning. GigaScience.

[100] Milani C., Casey E., Lugli G.A., Moore R., Kaczorowska J., Feehily C., Mangifesta M., Mancabelli L., Duranti S., Turroni F. (2018). Tracing mother-infant transmission of bacteriophages by means of a novel analytical tool for shotgun metagenomic datasets: METAnnotatorX. Microbiome.

[101] Sinno A., Baghdadi R., Narch R., El Rayes S., Tokajian S., Al Khoury C. (2025). Charting the virosphere: Computational synergies of AI and bioinformatics in viral discovery and evolution. J. Virol..

[102] Fu Y., Yu S., Li J., Lao Z., Yang X., Lin Z. (2024). DeepMineLys: Deep mining of phage lysins from human microbiome. Cell Rep..

[103] Pan J., Wang R., Ding W., Li Y., You Z., Huang Q., Wei D., Wang S., Sun Y. (2026). CM-PHI: Combining multi-hop attention graph neural network with sequence semantic analysis to predict phage-host interaction. Expert Syst. Appl..

[104] Zhu Q., Dai Q., He R., Huang J. (2022). PHIHNE: Predicting phage-host interaction through heterogeneous network embedding. Proceedings—2022 IEEE/WIC/ACM International Joint Conference on Web Intelligence and Intelligent Agent Technology (WI-IAT).

[105] Pan J., Wang R., Liu W., Wang L., You Z., Li Y., Duan Z., Huang Q., Feng J., Sun Y. (2025). Predicting phage-host interaction via hyperbolic Poincaré graph embedding and large-scale protein language technique. iScience.

[106] Chen Q., Zhao Z., Li M., Song W., Xiao M., Fang M. (2025). MoEPH: An adaptive fusion-based LLM for predicting phage-host interactions in health informatics. Front. Microbiol..

[107] Du Z.-H., Zhong J.-P., Liu Y., Li J.-Q. (2023). Prokaryotic virus host prediction with graph contrastive augmentation. PLoS Comput. Biol..

[108] Camejo P.Y., Rojas F., Ossa A., Hurtado R., Tichy D., Pieringer C., Pino M., Mora-Uribe P., Ulloa S., Norambuena R. (2025). A machine learning approach to predict strain-specific phage-host interactions. Sci. Rep..

[109] Xiao Z., Sun H., Wei A., Zhao W., Jiang X. (2025). A Novel Framework for Predicting Phage-Host Interactions via Host Specificity-Aware Graph Autoencoder. IEEE J. Biomed. Health Inform..

[110] Gonzales M.E.M., Ureta J.C., Shrestha A.M.S. (2023). Protein embeddings improve phage-host interaction prediction. PLoS ONE.

[111] Gonzales M.E.M., Ureta J.C., Shrestha A.M.S. (2024). PHIStruct: Improving phage-host interaction prediction at low sequence similarity settings using structure-aware protein embeddings. Bioinformatics.

[112] Li M.Q.C., Wang S., Lin S.R., Ting L.E.N., Wan Z.H., Xie G., Zhang J. (2026). Advantages and Limitations of AlphaFold in Structural Biology: Insights from Recent Studies. Protein J..

[113] Zhou F., Gan R., Zhang F., Ren C., Yu L., Si Y., Huang Z. (2022). PHISDetector: A Tool to Detect Diverse In Silico Phage-host Interaction Signals for Virome Studies. Genom. Proteom. Bioinform..

[114] Aggarwal S., Dhall A., Patiyal S., Choudhury S., Arora A., Raghava G.P.S. (2023). An ensemble method for prediction of phage-based therapy against bacterial infections. Front. Microbiol..

[115] Bodaka S., Kolliputi N. (2025). CoMPHI: A novel composite machine learning approach utilizing multiple feature representation to predict hosts of bacteriophages. Front. Bioinform..

[116] Pan J., You W., Lu X., Wang S., You Z., Sun Y. (2023). GSPHI: A novel deep learning model for predicting phage-host interactions via multiple biological information. Comput. Struct. Biotechnol. J..

[117] Malajczuk C.J., Vaitekenas A., Iszatt J.J., Stick S.M., Kicic A., Karpievitch Y.V. (2026). Towards accurate artificial intelligence models for strain-level phage-host prediction. Brief. Bioinform..

[118] Wei A., Xiao Z., Fu L., Zhao W., Jiang X. (2025). Predicting phage–host interactions via feature augmentation and regional graph convolution. Brief. Bioinform..

[119] Keith M., de la Torriente A.P., Chalka A., Vallejo-Trujillo A., McAteer S.P., Paterson G.K., Low A.S., Gally D.L. (2024). Predictive phage therapy for Escherichia coli urinary tract infections: Cocktail selection for therapy based on machine learning models. Proc. Natl. Acad. Sci. USA.

[120] Yang Y., Dufault-Thompson K., Yan W., Cai T., Xie L., Jiang X. (2024). Large-scale genomic survey with deep learning-based method reveals strain-level phage specificity determinants. GigaScience.

[121] Gomez de la Torre J.C., Frenkel A., Chavez-Lencinas C., Rendon A., Cáceres J.A., Alvarado L., Hueda-Zavaleta M. (2025). AI-based treatment recommendations enhance speed and accuracy in bacteremia management: A comparative study of molecular and phenotypic data. Life.

[122] Aleshkin A.V., Bochkareva S.S., Novikova L.I., Ershova O.N., Kiseleva I.A., Zubkova E.S., Anurova M.N., Rubalskii E.O., Zulkarneev E.R., Fedorova I.M. (2024). Personalized phagotherapy in the treatment of healthcare-associated infections caused by multidrug-resistant pathogens. Infektsionnye Bolezn..

[123] Aleshkin A.V., Shkoda A.S., Bochkareva S.S., Ershova O.N., Mitrokhin S.D., Kiseleva I.A., Zul’karneev E.R., Rubal’skiy E.O., Novikova L.I., Orlova O.E. (2017). Concept of individualized medicine based on personalized phage therapy for intensive care unit patients suffering from healthcare-associated infections. Infektsionnye Bolezn..

[124] Kasprzak H., Przybylski M., Fortuna W., Letkiewicz S., Rogóż P., Bubak B., Górski A., Międzybrodzki R. (2026). Analysis of pro- and anti-inflammatory gene response patterns in patients receiving phage therapy. Int. J. Mol. Sci..

[125] Scarlata G.G.M., Belančić A., Štimac D., Fajkić A., Meštrović T., Abenavoli L. (2026). Bacteriophage therapy against Shigella spp.: A precision antimicrobial strategy. Antibiotics.

[126] Molina F., Menor-Flores M., Vega-Rodríguez M.A. (2025). SocialViruses: Integrating quantitative phage–bacteria and phage–phage interaction networks for rational cocktail design. Bioinform. Adv..

[127] Molina F., Simancas A., Ramírez M., Tabla R., Roa I., Rebollo J.E. (2021). A new pipeline for designing phage cocktails based on phage-bacteria infection networks. Front. Microbiol..

[128] Yoo S., Lee K.-M., Kim N., Vu T.N., Abadie R., Yong D. (2024). Designing phage cocktails to combat the emergence of bacteriophage-resistant mutants in multidrug-resistant Klebsiella pneumoniae. Microbiol. Spectr..

[129] Kim M.K., Chen Q., Echterhof A., Pennetzdorfer N., McBride R.C., Banaei N., Burgener E.B., Milla C.E., Bollyky P.L. (2024). A blueprint for broadly effective bacteriophage-antibiotic cocktails against bacterial infections. Nat. Commun..

[130] Zhao M., Li H., Gan D., Wang M., Deng H., Yang Q.E. (2024). Antibacterial effect of phage cocktails and phage-antibiotic synergy against pathogenic Klebsiella pneumoniae. mSystems.

[131] Costa P., Pereira C., Romalde J.L., Almeida A. (2025). From isolation to application: Designing a multi-target phage cocktail for bivalve safety. Microorganisms.

[132] Cha J.S., Kim K., You H.J., Kim D., Park H.H., Heo S., Kim C.O., Jin B.H., Yong D., Chae D. (2025). Model-informed development of bacteriophage therapy: Bridging in vitro and in vivo efficacy against multidrug-resistant Pseudomonas aeruginosa. mSystems.

[133] Lee C.K., Lee H.J., Jeong S.H., Lee S.J. (2025). Precision targeting of genetic variations in mixed bacterial cultures using CRISPR-Cas12a-programmed λ phages. Front. Microbiol..

[134] Galgat H., Pouya N., Tam V.H., Nikolaou M. (2026). Using machine learning to design effective antimicrobial dosing regimens. Comput. Chem. Eng..

[135] Mukhopadhyay S., Zhang X., To K.K.W., Liu Y., Bai C., Leung S.S.Y. (2026). Treatment sequences of a phage and colistin combination trigger different evolution pathways of a multidrug-resistant Acinetobacter baumannii yielding distinct treatment outcomes. Int. J. Antimicrob. Agents.

[136] Smith N.M., Nguyen T.D., Chin W.H., Sanborn J.T., de Souza H., Ho B.M., Luong T., Roach D.R. (2023). A mechanism-based pathway toward administering highly active N-phage cocktails. Front. Microbiol..

[137] Nguyen T.D., Sanborn J.T., Ho B.M., Luong T., Wood T.D., Chen L., Roach D.R., Smith N.M. (2026). Pharmacodynamic individualization of phage therapy against a KPC-5-producing Pseudomonas aeruginosa. JAC-Antimicrob. Resist..

[138] Lee J.-W., Kim J., Kim S. (2025). Phage-antibiotic synergy review: Mechanisms, applications, and future prospects. J. Bacteriol. Virol..

[139] Ren X., Lu R., You X., Zhu R., Li Y. (2025). Recent advances in phage-antibiotic combination therapy for bacterial infections. Acta Microbiol. Sin..

[140] Fatima R., Hynes A.P. (2025). Phage-antibiotic combinations for Pseudomonas: Successes in the clinic and in vitro tenuously connected. Microb. Biotechnol..

[141] Selim S. (2026). Chemical and biological strategies to disrupt biofilms: A new era in infectious disease management and antimicrobial resistance control. BioResources.

[142] Karamlou S., Aghajani E., Familsamavati M., Azad N.F., Arayesh S., Mohsenipour Z. (2025). Bacterial resistance to phage therapy: Mechanisms and strategies to overcome it. Curr. Mol. Pharmacol..

[143] Derollez E., Roson-Calero N., Rouzé P., Dedieu-Berne A., Ballesté-Delpierre C., Fraikin N., Iorga B.I., Huang T.-D., Bigot S., Vila J. (2026). Specific killing and resensitization of pathogenic Escherichia coli strains carrying bla CTX-M-15 β-lactamase using targeted-antibacterial-plasmids (TAPs). Nucleic Acids Res..

[144] Huan Y.W., Torraca V., Brown R., Fa-Arun J., Miles S.L., Oyarzún D.A., Mostowy S., Wang B. (2023). P1 bacteriophage-enabled delivery of CRISPR-Cas9 antimicrobial activity against Shigella flexneri. ACS Synth. Biol..

[145] Tsolakidou P.J. (2025). CRISPR–Cas systems against carbapenem resistance: From proof-of-concept to clinical translation. Front. Microbiol..

[146] Reuter A., Hilpert C., Dedieu-Berne A., Lematre S., Gueguen E., Launay G., Bigot S., Lesterlin C. (2021). Targeted-antibacterial-plasmids (TAPs) combining conjugation and CRISPR/Cas systems achieve strain-specific antibacterial activity. Nucleic Acids Res..

[147] Citorik R.J., Mimee M., Lu T.K. (2014). Sequence-specific antimicrobials using efficiently delivered RNA-guided nucleases. Nat. Biotechnol..

[148] Ataee S., Brochet X., Peña-Reyes C.A. (2022). Bacteriophage genetic edition using LSTM. Front. Bioinform..

[149] Wang R., Shu X., Zhao H., Xue Q., Liu C., Wu A., Cheng F., Wang L., Zhang Y., Feng J. (2023). Associate toxin-antitoxin with CRISPR-Cas to kill multidrug-resistant pathogens. Nat. Commun..

[150] Yoon B., Kim J.A., Kang Y.K. (2026). CRISPR–Cas-mediated reprogramming strategies to overcome antimicrobial resistance. Pharmaceutics.

[151] Gupta D.P., Bondre S.R., Khade P.S., Harde S., Nasare S.O., Pardhi P.D., Wadher K. (2026). CRISPR-compatible biomaterials: A new frontier in gene-responsive drug delivery. Int. J. Drug Deliv. Technol..

[152] Shchukina E.I., Mazunin I.O., Eremin I.I., Moskalev A.A. (2025). Gene therapy techniques and delivery methods (review). Sovrem. Tehnol. Med..

[153] Liu C.J.S., Wang S.-B., Su P.-Y., Hsieh Y.-P., Wang H.-Y. (2026). Microfluidic electroporation for drug and gene delivery: Driving innovation from single-cell precision to high-throughput preclinical and therapeutic platforms. Adv. Drug Deliv. Rev..

[154] Shao B., Yan J. (2024). A long-context language model for deciphering and generating bacteriophage genomes. Nat. Commun..

[155] Lenneman B.R., Fernbach J., Loessner M.J., Lu T.K., Kilcher S. (2021). Enhancing phage therapy through synthetic biology and genome engineering. Curr. Opin. Biotechnol..

[156] Deng T., Ge X., Wang J. (2026). Structures of λ-like phage A8 tail tip bound to OmpC provide insight into receptor recognition. Structure.

[157] Ritter S.C., Hackel B.J. (2019). Validation and stabilization of a prophage lysin of Clostridium perfringens by using yeast surface display and coevolutionary models. Appl. Environ. Microbiol..

[158] Ma W., Lei Y., Wang L., Chen Y., Ma M., Chen X. (2026). Bacteriophage lysins as programmable antimicrobials: Mechanisms, engineering, and translational advances. Probiotics Antimicrob. Proteins.

[159] Almusallam N., Hayat M. (2026). Explainable AI for secure and accurate prediction of bacteriophage virion proteins using NLP descriptors and transformer-guided ideal proximity matrix reconstruction. Chemom. Intell. Lab. Syst..

[160] Xie Y., Pan J., Li D., Wang Q., Sun Y., Wang S. (2026). MVPHI: A multi-view learning framework for predicting complex microbial interactions. Sci. Rep..

[161] Nazir A., Xu X., Liu Y., Chen Y. (2023). Phage endolysins: Advances in the world of food safety. Cells.

[162] Kong C., Huang L.-B., Yang M.-F., Yue N.-N., Luo D., Zhang Y., Tian C.-M., Song Y., Wei D.-R., Shi R.-Y. (2025). Microbiome engineering: Unlocking therapeutic potential in inflammatory bowel disease. Front. Microbiol..

[163] Ali Z., Rehman S., Naseem S. (2025). Bacteriophages as therapeutic and biotechnological agents: Host interactions, immune modulation, and engineering strategies. PHAGE Ther. Appl. Res..

[164] Hsu C.-Y., Polatova D., Hamad R.H., Patel P.N., Akram M., Singh G., Arora V., Nayak P.P., Kadhem M., Hamzah H.F. (2026). Phage therapy in cancer treatment: Mechanisms, emerging innovations, and translational progress. Crit. Rev. Oncol. Hematol..

[165] Alessa O., Aiba Y., Arbaah M., Hidaka Y., Watanabe S., Miyanaga K., Wannigama D.L., Cui L. (2025). Synthetic and functional engineering of bacteriophages: Approaches for tailored bactericidal, diagnostic, and delivery platforms. Molecules.

[166] Rezaei M., Jalali A., Al-Azzawi D.H.S. (2025). Engineered bacteriophages: Advances in phage genome redesign strategies for therapeutic and environmental applications. Protein Pept. Lett..

[167] Gladue D.P., O’Mahony A. (2025). CRISPR treatments for AI-designed synthetic viruses: Rapid programmable countermeasures for emerging and engineered viruses. Viruses.

[168] Yu X., Zhang J., Li X., Li G., Lu X., Shi Y., Lin W., Wang X., Zhang W., Tong Y. (2026). A review of phage therapy for drug-resistant Pseudomonas aeruginosa infections. Microbiol. Res..

[169] Schwarz C., Mathieu J., Laverde Gomez J.A., Yu P., Alvarez P.J.J. (2022). Renaissance for phage-based bacterial control. Environ. Sci. Technol..

[170] Ali J., Rafiq Q., Ratcliffe E. (2019). A scaled-down model for the translation of bacteriophage culture to manufacturing scale. Biotechnol. Bioeng..

[171] Jha D.K., Archana S., Elyasi Z. (2025). Bioprocess optimization strategies: Enhancing efficiency and yield in bio-manufacturing. Industrial Applications for Bioprocessing and Biomanufacturing.

[172] Konopacki M., Grygorcewicz B., Gliźniewicz M., Miłek D., Kordas M., Rakoczy R. (2022). Dynamic modelling of bacteriophage production process. Chem. Process Eng.-Inz. Chem. I Proces..

[173] Luan C., Liu W., Yao Y., Meng J., Wang D. (2026). Emerging applications of artificial intelligence in microbial-based sugar alcohol and organic acid production: Recent advances. Food Biosci..

[174] Tarafdar A., Das A., Bhupender, Singh B. (2025). Application of artificial intelligence and machine learning in solid-state fermentation processes. Current Advances in Solid-State Fermentation: Current Developments in Biotechnology and Bioengineering.

[175] Al-Hindi R.R., Teklemariam A.D., Alharbi M.G., Alotibi I., Azhari S.A., Qadri I., Alamri T., Harakeh S., Applegate B.M., Bhunia A.K. (2022). Bacteriophage-Based Biosensors: A Platform for Detection of Foodborne Bacterial Pathogens from Food and Environment. Biosensors.

[176] Haq I.U., Rahim K., Maryam S., Paker N.P. (2025). Bacteriophage-based biosensors technology: Materials, fabrications, efficiencies and shortcomings. Biotechnol. Rep..

[177] Heras J.Y., Pallarola D., Battaglini F. (2010). Electronic tongue for simultaneous detection of endotoxins and other contaminants of microbiological origin. Biosens. Bioelectron..

[178] Ni Y., Wang Y., Meng X., Lin J., Wang X. (2026). QDs-based SERS technology: Breakthroughs and prospects from biosensing to food safety. Anal. Sens..

[179] Jiang S., Noh J., Park C., Smith A.D., Abbott N.L., Zavala V.M. (2021). Using machine learning and liquid crystal droplets to identify and quantify endotoxins from different bacterial species. Analyst.

[180] Dhiman B., Kumar S., Singh S.K., Arya V., Ratnaparkhi A. (2026). AI and blockchain for secure semiconductor supply chain transparency. AI-Driven Hardware Security: Architectures, Chips, and Trust.

[181] Nallasamy V., Sughumaran C.T., Krishnan V.L., Singh S. (2025). Artificial intelligence-powered detection systems for antibiotic residues in food and the environment: A mini review with special focus on milk products and environmental matrices analysis. Recent Adv. Food Nutr. Agric..

[182] Kumar R., Chaudhry V., Prakash O. (2022). Editorial: Multi-omics profiling of unique niches to reveal the microbial and metabolite composition. Front. Microbiol..

[183] Tarlak F. (2023). The Use of Predictive Microbiology for the Prediction of the Shelf Life of Food Products. Foods.

[184] Wendling C.C., Vasse M., Wielgoss S. (2025). Phage quest: A beginner’s guide to explore viral diversity in the prokaryotic world. Brief. Bioinform..

[185] Parrondo-Pizarro R., Lanini J., Rodríguez-Pérez R. (2026). Uncertainty Quantification in Molecular Machine Learning for Property Predictions under Data Shifts. J. Chem. Inf. Model..

[186] Sharma J., Goel P. (2025). The use of AI for phenotype-genotype mapping. Methods Mol. Biol..

[187] Okubo Y., Zhang Y.-Z., Imoto S. (2025). Multi-instance contrastive learning with binomial k-mers for phage host interaction prediction. 2025 13th International Conference on Bioinformatics and Computational Biology (ICBCB).

[188] Tange R.I., Rasmussen M.A., Taira E., Bro R. (2017). Benchmarking support vector regression against partial least squares regression and artificial neural network: Effect of sample size on model performance. J. Near Infrared Spectrosc..

[189] Ito T., Nguyen T.D., Saito Y., Kurumida Y., Nakazawa H., Kawada S., Nishi H., Tsuda K., Kameda T., Umetsu M. (2023). Selection of target-binding proteins from the information of weakly enriched phage display libraries by deep sequencing and machine learning. mAbs.

[190] Kawada S., Kurumida Y., Ito T., Nguyen T.D., Nishi H., Nakazawa H., Saito Y., Kameda T., Tsuda K., Umetsu M. (2025). Discovery and affinity maturation of antibody fragments from an unfavorably enriched phage display selection by deep sequencing and machine learning. J. Biosci. Bioeng..

[191] Liew B.X.W., Pfisterer F., Rügamer D., Zhai X. (2024). Strategies to optimise machine learning classification performance when using biomechanical features. J. Biomech..

[192] Lamouadene H., Kassaoui M.E., Yadari M.E., Kenz A.E., Benyoussef A. (2025). Exploring modeling techniques for predicting band gaps of Doped-ZnO: A Machine learning approach. Chem. Phys..

[193] Rajaraman S., Antani S. (2020). Weakly labeled data augmentation for deep learning: A study on COVID-19 detection in chest X-rays. Diagnostics.

[194] Papadopoulos D., Karali G., Karalis V.D. (2024). Bioequivalence studies of highly variable drugs: An old problem addressed by artificial neural networks. Appl. Sci..

[195] Liu Y., Yang Y., Lu H., Cui J., Chen X., Ma P., Zhong W., Zhao Y. (2025). Extracting true virus SERS spectra and augmenting data for improved virus classification and quantification. ACS Sens..

[196] Zhao Z., Chen Q., Li M., Song W., Xiao M., Fang M. (2026). Sequence-based deep learning ensemble for targeted phage–host interaction prediction. Data Science.

[197] Danilevicz M.F., Upadhyaya S.R., Batley J., Bennamoun M., Bayer P.E., Edwards D. (2025). Understanding plant phenotypes in crop breeding through explainable AI. Plant Biotechnol. J..

[198] Hammouda N., Mahfoudh M., Boukadi K., Salameh K., Chbeir R. (2026). Connecting AI, explainability and semantic in animal applications: A scoping review. Expert Syst..

[199] Nilsson A.S. (2014). Phage therapy-constraints and possibilities. Upsala J. Med. Sci..

[200] Kłopot A., Zakrzewska A., Lecion D., Majewska J.M., Harhala M.A., Lahutta K., Kazmierczak Z., Łaczmanski Ł., Kłak M., Dabrowska K. (2017). Real-Time qPCR as a method for detection of antibody-neutralized phage particles. Front. Microbiol..

[201] Petrovic Fabijan A., Khalid A., Maddocks S., Ho J., Gilbey T., Sandaradura I., Lin R.C.Y., Ben Zakour N., Venturini C., Bowring B. (2020). Phage therapy for severe bacterial infections: A narrative review. Med. J. Aust..

[202] Suh G.A., Patel R. (2023). Clinical phage microbiology: A narrative summary. Clin. Microbiol. Infect..

[203] Nilsson A.S. (2019). Pharmacological limitations of phage therapy. Upsala J. Med. Sci..

[204] Rao G.G., Vallé Q., Mahadevan R., Sharma R., Barr J.J., Van Tyne D. (2025). Crossing the chasm: How to approach translational pharmacokinetic–pharmacodynamic modeling of phage dosing. Clin. Pharmacol. Ther..

[205] Weld R.J., Butts C., Heinemann J.A. (2004). Models of phage growth and their applicability to phage therapy. J. Theor. Biol..

[206] Pandolfo M., Telatin A., Lazzari G., Adriaenssens E.M., Vitulo N. (2022). MetaPhage: An automated pipeline for analyzing, annotating, and classifying bacteriophages in metagenomics sequencing data. mSystems.

[207] Amgarten D., Iha B.K.V., Piroupo C.M., Da Silva A.M., Setubal J.C. (2022). VHULK, a new tool for bacteriophage host prediction based on annotated genomic features and neural networks. PHAGE Ther. Appl. Res..

[208] Zielezinski A., Barylski J., Karlowski W.M. (2021). Taxonomy-aware, sequence similarity ranking reliably predicts phage–host relationships. BMC Biol..

[209] Gaborieau B., Vaysset H., Tesson F., Charachon I., Dib N., Bernier J., Dequidt T., Georjon H., Clermont O., Hersen P. (2024). Prediction of strain level phage–host interactions across the Escherichia genus using only genomic information. Nat. Microbiol..

[210] Lieto K., Skopek R., Lewicka A., Stelmasiak M., Klimaszewska E., Zelent A., Szymański Ł., Lewicki S. (2022). Looking into the eyes—In vitro models for ocular research. Int. J. Mol. Sci..

[211] Bröcker F., Willy C. (2025). Potential of bacteriophage therapy in Germany: Evidence and clinical relevance [Potenziale der Bakteriophagentherapie in Deutschland: Evidenzlage und klinische Relevanz]. Bundesgesundheitsblatt-Gesundheitsforschung-Gesundheitsschutz.

[212] Zillmann H. (2025). Medical ethical challenges of phage therapy—Informed consent, study design and therapeutic trial [Medizinethische Herausforderungen der Phagentherapie—Informierte Einwilligung, Studiendesign und Heilversuch]. Bundesgesundheitsblatt-Gesundheitsforschung-Gesundheitsschutz.

[213] Philipson C.W., Voegtly L.J., Lueder M.R., Long K.A., Rice G.K., Frey K.G., Biswas B., Cer R.Z., Hamilton T., Bishop-Lilly K.A. (2018). Characterizing phage genomes for therapeutic applications. Viruses.

[214] Chung K.M., Nang S.C., Tang S.S. (2023). The safety of bacteriophages in treatment of diseases caused by multidrug-resistant bacteria. Pharmaceuticals.

[215] Mukala P. (2026). Ethical implications of black box AI models in high-stakes applications. Information System Design: AI and ML Applications.

[216] Ravindran S.K., Kot E., Nah F.F.-H. (2026). Can we read AI’s mind? A quest for transparency. HCI International 2025—Late Breaking Papers.

[217] Atoum I. (2025). Revolutionizing AI governance: Addressing bias and ensuring accountability through the holistic AI governance framework. Int. J. Adv. Comput. Sci. Appl..

[218] Ozmen B.B., Taub P.J. (2026). Ethical and regulatory considerations for artificial intelligence adoption in craniofacial surgery. J. Craniofacial Surg..

[219] Luckner S., Lauer W. (2025). Regulatory classification of AI-enabled products for medical use on the basis of the EU AI Act and MDR/IVDR [Regulatorische Einordnung KI-basierter Produkte für die medizinische Anwendung auf Basis von EU AI Act und MDR/IVDR]. Bundesgesundheitsblatt—Gesundheitsforschung—Gesundheitsschutz.

[220] Loucif S., Sharma R., Kshetri N., Zahid A. (2025). From design to decommissioning: TAFES framework for responsible AI. Law. Ethics Technol..

[221] Martinho D., Sobreiro P., Domingues A., Martinho F., Nogueira N. (2026). Ethical responsibility in medical AI: A semi-systematic thematic review and multilevel governance model. Healthcare.

[222] Zangana H.M., Omar M., Al-Karaki J.N. (2025). Data privacy and security standards in AI-powered scientific research. Ensuring Secure and Ethical STM Research in the AI Era.

[223] Adeyinka T.I., Adeyinka K.I. (2025). Reducing the carbon footprint in healthcare cybersecurity threats in AI-powered healthcare systems data privacy in AI-driven diagnostics. AI-Driven Healthcare Cybersecurity and Privacy.

[224] Palacio A.L., Reyes Román J.F., Pastor O. BETTER: Better rEal-world healTh-DaTa distributEd analytics research platform. Proceedings of the CEUR Workshop Proceedings.

[225] Liu S., Guo L.R. (2024). Data Ownership in the AI-Powered Integrative Health Care Landscape. JMIR Med. Inform..

[226] Goel A., Prabha C. (2026). Federated learning: A paradigm shift in healthcare data privacy. Integrating Cloud, Fog, and Edge Computing in Healthcare: Federated Learning and Blockchain Approaches.

[227] Taddese A.A., Addis A.C., Tam B.T. (2025). Data stewardship and curation practices in AI-based genomics and automated microscopy image analysis for high-throughput screening studies: Promoting robust and ethical AI applications. Hum. Genom..

[228] Mehrtabar S., Marey A., Desai A., Saad A.M., Desai V., Goñi J., Pal B., Umair M. (2025). Ethical considerations in patient privacy and data handling for AI in cardiovascular imaging and Radiology. J. Imaging Inform. Med..

[229] Murdoch B. (2021). Privacy and artificial intelligence: Challenges for protecting health information in a new era. BMC Med. Ethics.

